# Supplementary material for: Profiling of the TCRβ repertoire in non-model species using high-throughput sequencing
Source: Sci Rep. 2018 Aug 2;8:11613. doi: 10.1038/s41598-018-30037-0 (PMC6072738; doi:10.1038/s41598-018-30037-0)
Supplement: Supplementary file 1 — Supplementary Information [file 41598_2018_30037_MOESM1_ESM.pdf]

Supplementary Information for:

Title: Profiling of the TCRβ repertoire in non-model species using high-throughput sequencing.

[1*]Magdalena Migalska, [1,2]Alvaro Sebastian, [1]Jacek Radwan

[1]Evolutionary Biology Group, Faculty of Biology, Adam Mickiewicz University, ul. Umultowska 89, 61-614 Poznan, Poland (http://evobiolab.biol.amu.edu.pl)

[2]Instituto Aragonés de Empleo (INAEM), c/ Royo Villanova 1, 50007 Zaragoza, Spain

*To whom correspondence should be addressed. Email: magdalena.migalska@amu.edu.pl

<u>The file contains:</u>

Supplementary Figures S1-S10

Supplementary Tables S1-S7

Supplementary Files S1-S3:

File S1 – Molecular protocol for T-cell receptor cDNA library preparation by 5'RACE for deep HTS profiling.

File S2 – Partial, consensus sequences of the bank vole J genes identified in this study.

File S3 – Partial, consensus sequences of the bank vole V genes identified in this study.

Supplementary Figures

Supplementary Figure S1. Schematic representation of the 5′ RACE procedure for library construction. In the first step, the mRNA of TCRβ chains is reverse transcribed with a primer matching the 5′ end of the constant region of a TCR, and a universal adaptor is added at the 5′ end of the cDNA. For quantitative analysis, the universal adaptor is modified to contain a unique molecular identifier (UMI). cDNA is amplified with one primer pair: the forward primer matching universal adaptor sequence is added to the 5′ end during reverse transcription, and the reverse primer is in the constant region. Forward and reverse primers used in 5′ RACE are represented as black arrows; RACE 5′ adaptor as a grey bar; modified RACE 5′ adaptor as a grey bar with a multi-coloured box representing UMI; and the sequence covered by Illumina sequencing as a thick, grey arrow. Location of the CDR3 region is shaded in grey.

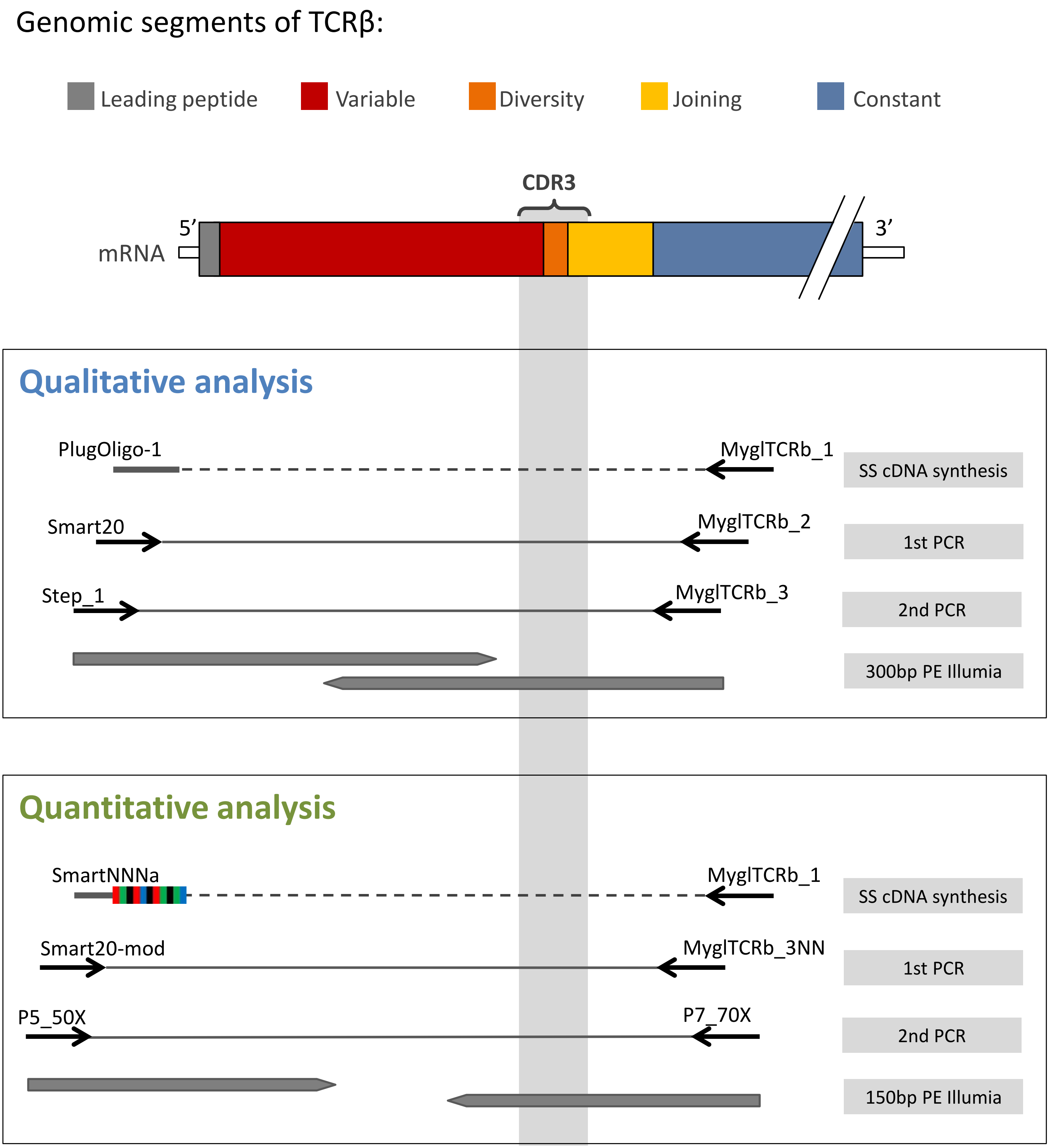
Genomic segments of TCRβ:
Leading peptide
Variable
Diversity
Joining
Constant
CDR3
5′
3′
mRNA
Qualitative analysis
PlugOligo-1
MyglTCRb_1
SS cDNA synthesis
Smart20
MyglTCRb_2
1st PCR
Step_1
MyglTCRb_3
2nd PCR
300bp PE Illumia
Quantitative analysis
SmartNNNa
MyglTCRb_1
SS cDNA synthesis
Smart20-mod
MyglTCRb_3NN
1st PCR
P5_50X
P7_70X
2nd PCR
150bp PE Illumia

Supplementary Figure S2. **Neighbour-joining phylogenetic tree of 17 nucleotide sequences of bank vole TCRβ J alleles retrieved in the present study and the mouse reference.** An analogical tree was used to name the bank vole alleles, with numbering corresponding to murine orthologues. Bootstrap values based on 1000 replicates are shown. Branch lengths are in units of evolutionary distances (the number of base substitutions per site). The full list of mouse reference sequences with accession numbers is available in Supplementary Table S1, sequences of bank vole genes identified in this study are available in Supplementary File S2. Orange circles mark the mouse reference sequences; red circles denote murine sequence in an open reading frame but with alterations in regulatory elements or splice sites (ORF); and blue circles mark the bank vole sequences. Numbers after asterisks refer to the allele name.

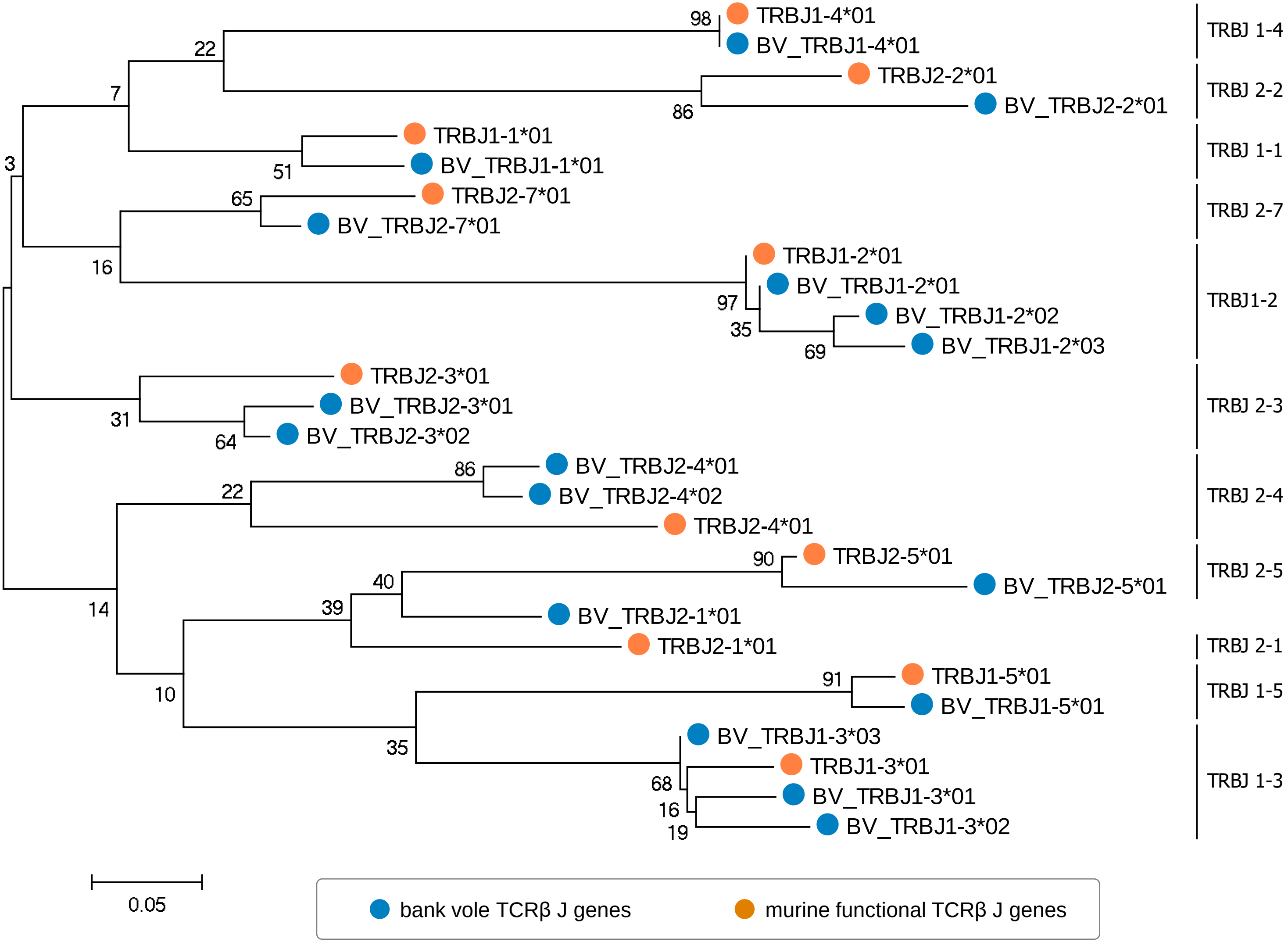

98
TRBJ1-4*01
BV_TRBJ1-4*01
TRBJ 1-4
22
TRBJ2-2*01
BV_TRBJ2-2*01
86
TRBJ 2-2
7
TRBJ1-1*01
BV_TRBJ1-1*01
51
TRBJ 1-1
3
65
TRBJ2-7*01
BV_TRBJ2-7*01
TRBJ 2-7
16
TRBJ1-2*01
BV_TRBJ1-2*01
97
35
BV_TRBJ1-2*02
BV_TRBJ1-2*03
69
TRBJ1-2
TRBJ2-3*01
BV_TRBJ2-3*01
31
64
BV_TRBJ2-3*02
TRBJ 2-3
86
BV_TRBJ2-4*01
22
BV_TRBJ2-4*02
TRBJ2-4*01
TRBJ 2-4
90
TRBJ2-5*01
40
BV_TRBJ2-5*01
TRBJ 2-5
39
14
BV_TRBJ2-1*01
TRBJ2-1*01
TRBJ 2-1
91
TRBJ1-5*01
10
BV_TRBJ1-5*01
TRBJ 1-5
BV_TRBJ1-3*03
35
TRBJ1-3*01
68
BV_TRBJ1-3*01
16
19
BV_TRBJ1-3*02
TRBJ 1-3
0.05
bank vole TCRβ J genes
murine functional TCRβ J genes

Supplementary Figure S3. Neighbour-joining phylogenetic tree of 116 nucleotide sequences of bank vole TCRβ V alleles retrieved in the present study and the mouse reference. An analogical tree was used to name the bank vole alleles with numbering corresponding to murine orthologues. Bootstrap values greater than 75, based on 1000 replicates, are shown. Branch lengths are in units of evolutionary distances (the number of base substitutions per site). A full list of mouse reference sequences with accession numbers is available in Supplementary Table S1; and sequences of bank vole genes identified in this study are available in Supplementary File S3. Orange circles mark the mouse reference sequences; red circles denote murine pseudogenes (P) or sequences in an open reading frame but with alterations in regulatory elements or splice sites (ORF); and blue circles mark the bank vole sequences. Number after asterisks refer to the allele name.

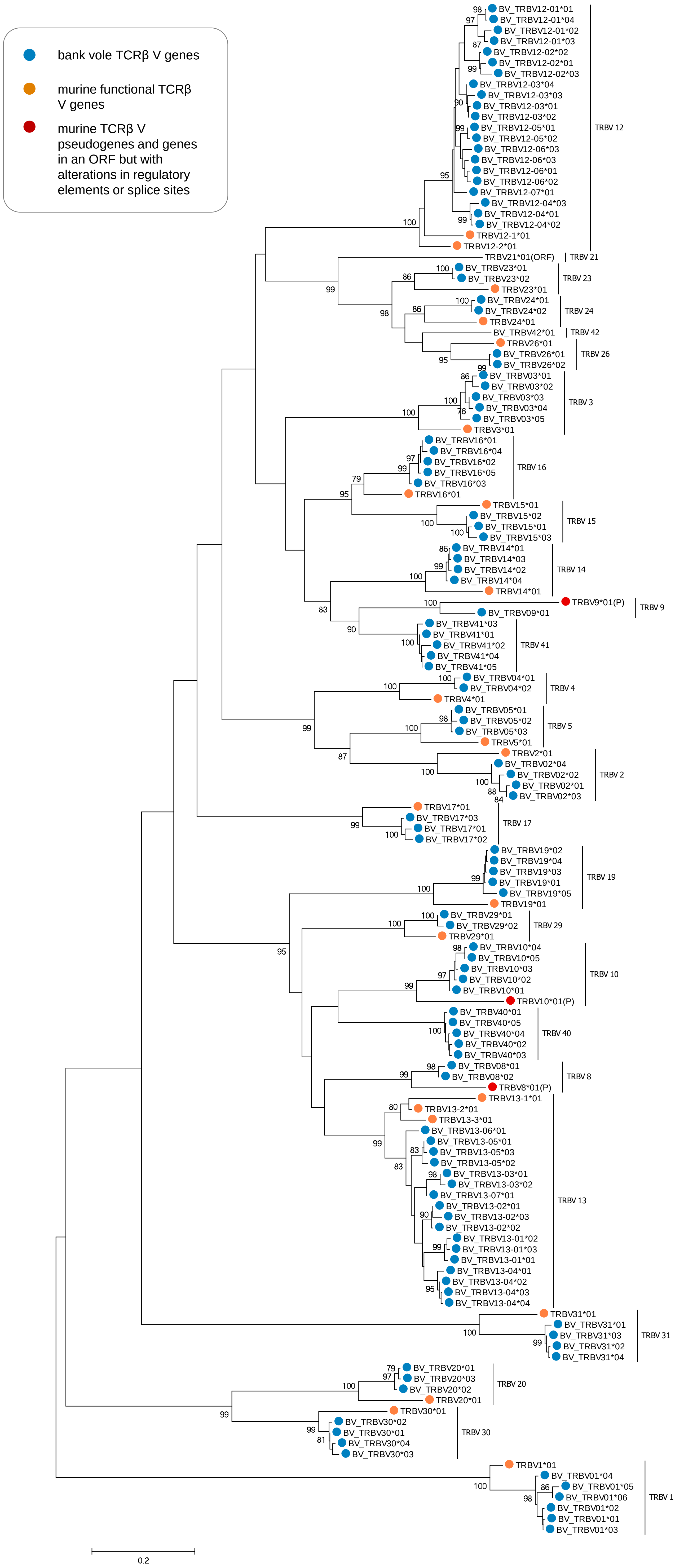
bank vole TCRβ V genes
murine functional TCRβ V genes
murine TCRβ V pseudogenes and genes in an ORF but with alterations in regulatory elements or splice sites
TRBV 12
TRBV 21
TRBV 23
TRBV 24
TRBV 42
TRBV 26
TRBV 3
TRBV 16
TRBV 15
TRBV 14
TRBV 9
TRBV 41
TRBV 4
TRBV 5
TRBV 2
TRBV 17
TRBV 19
TRBV 29
TRBV 10
TRBV 40
TRBV 8
TRBV 13
TRBV 31
TRBV 20
TRBV 30
TRBV 1
0.2

Supplementary Figure S4. Presence of identified BV_TRBJ alleles in seven analysed bank voles. An allele present in a given individual is marked as a grey box.

Gene and allele name	S1	S2	S3	S4	S5	S6	S7
BV_TRBJ1-1*01	■	■	■	■	■	■	■
BV_TRBJ1-2*01	■	■	■	■	■	■	■
BV_TRBJ1-2*02		■			■		
BV_TRBJ1-2*03						■	
BV_TRBJ1-3*01	■	■		■		■	
BV_TRBJ1-3*02		■	■		■		
BV_TRBJ1-3*03				■	■	■	■
BV_TRBJ1-4*01	■	■	■	■	■	■	■
BV_TRBJ1-5*01	■	■	■	■	■	■	■
BV_TRBJ2-1*01	■	■	■	■	■	■	■
BV_TRBJ2-2*01	■	■	■		■	■	
BV_TRBJ2-3*01	■	■	■	■	■	■	■
BV_TRBJ2-3*02	■						
BV_TRBJ2-4*01	■		■				
BV_TRBJ2-4*02		■		■	■	■	■
BV_TRBJ2-5*01	■	■	■	■	■	■	■
BV_TRBJ2-7*01	■	■	■	■	■	■	■

Supplementary Figure S5. Presence of identified BV_TRBV alleles in seven analysed bank voles. Allele present in a given individual is marked as a grey box.

Gene and allele name	S1	S2	S3	S4	S5	S6	S7
BV_TCRBV01*01	■			■			
BV_TCRBV01*02		■	■				
BV_TCRBV01*03					■	■	
BV_TCRBV01*04						■	
BV_TCRBV01*05			■	■	■		
BV_TCRBV01*06							■
BV_TCRBV02*01	■	■	■	■			
BV_TCRBV02*02			■			■	
BV_TCRBV02*03				■	■		
BV_TCRBV02*04							■
BV_TCRBV03*01	■		■	■			
BV_TCRBV03*02			■			■	
BV_TCRBV03*03				■	■		
BV_TCRBV03*04						■	
BV_TCRBV03*05							■
BV_TCRBV04*01	■	■	■	■			
BV_TCRBV04*02				■	■	■	■
BV_TCRBV05*01	■	■	■	■			
BV_TCRBV05*02				■	■	■	
BV_TCRBV05*03							■
BV_TCRBV08*01	■	■	■	■			
BV_TCRBV08*02				■	■	■	■
BV_TCRBV09*01	■	■	■	■	■	■	■
BV_TCRBV10*01	■			■			
BV_TCRBV10*02		■	■				
BV_TCRBV10*03			■			■	
BV_TCRBV10*04					■		■
BV_TCRBV10*05						■	
BV_TCRBV12-01*01	■	■	■				
BV_TCRBV12-01*02	■			■			
BV_TCRBV12-01*03				■	■	■	■
BV_TCRBV12-01*04						■	
BV_TCRBV12-02*01	■			■			
BV_TCRBV12-02*02		■	■			■	
BV_TCRBV12-02*03					■	■	■
BV_TCRBV12-03*01	■			■			
BV_TCRBV12-03*02		■	■				
BV_TCRBV12-03*03				■	■	■	■
BV_TCRBV12-03*04						■	
BV_TCRBV12-04*01	■			■	■	■	■
BV_TCRBV12-04*02	■		■			■	
BV_TCRBV12-04*03	■	■	■				
BV_TCRBV12-05*01	■			■	■	■	■
BV_TCRBV12-05*02						■	
BV_TCRBV12-06*01	■			■			
BV_TCRBV12-06*02		■	■				
BV_TCRBV12-06*03				■	■	■	■
BV_TCRBV12-06*04						■	
BV_TCRBV12-07*01				■	■		■
BV_TCRBV13-01*01	■		■	■			
BV_TCRBV13-01*02		■	■			■	
BV_TCRBV13-01*03				■	■	■	■
BV_TCRBV13-02*01	■			■			
BV_TCRBV13-02*02		■	■				
BV_TCRBV13-02*03			■	■	■	■	■
BV_TCRBV13-03*01		■	■				
BV_TCRBV13-03*02					■	■	■
BV_TCRBV13-04*01	■			■			

Gene and allele names	S1	S2	S3	S4	S5	S6	S7
BV_TCRBV13-04*02		■	■				
BV_TCRBV13-04*03				■	■	■	■
BV_TCRBV13-04*04						■	
BV_TCRBV13-05*01			■	■	■	■	■
BV_TCRBV13-05*02				■			
BV_TCRBV13-05*03						■	
BV_TCRBV13-06*01	■	■	■	■			
BV_TCRBV13-07*01	■		■	■	■	■	■
BV_TCRBV14*01		■	■				
BV_TCRBV14*02				■	■	■	■
BV_TCRBV14*03						■	
BV_TCRBV14*04	■			■			
BV_TCRBV15*01	■	■	■				
BV_TCRBV15*02				■	■	■	■
BV_TCRBV15*03						■	
BV_TCRBV16*01	■			■			
BV_TCRBV16*02		■	■				
BV_TCRBV16*03			■				
BV_TCRBV16*04					■		■
BV_TCRBV16*05						■	
BV_TCRBV17*01	■	■	■	■			
BV_TCRBV17*02				■	■	■	■
BV_TCRBV17*03						■	
BV_TCRBV19*01	■			■			
BV_TCRBV19*02		■	■				
BV_TCRBV19*03			■				
BV_TCRBV19*04				■	■	■	■
BV_TCRBV19*05						■	
BV_TCRBV20*01	■			■		■	
BV_TCRBV20*02		■	■				
BV_TCRBV20*03			■	■	■	■	■
BV_TCRBV23*01	■	■	■	■	■	■	■
BV_TCRBV23*02						■	
BV_TCRBV24*01	■	■	■	■	■	■	■
BV_TCRBV24*02						■	
BV_TCRBV26*01	■	■		■	■		
BV_TCRBV26*02					■	■	■
BV_TCRBV29*01	■			■	■	■	■
BV_TCRBV29*02		■	■			■	
BV_TCRBV30*01	■	■	■	■			
BV_TCRBV30*02			■				
BV_TCRBV30*03				■	■	■	■
BV_TCRBV30*04						■	
BV_TCRBV31*01	■			■	■	■	■
BV_TCRBV31*02		■	■				
BV_TCRBV31*03					■		
BV_TCRBV31*04						■	
BV_TCRBV40*01	■			■			
BV_TCRBV40*02		■	■				
BV_TCRBV40*03			■				
BV_TCRBV40*04				■	■	■	■
BV_TCRBV40*05						■	
BV_TCRBV41*01	■	■		■			
BV_TCRBV41*02		■	■				
BV_TCRBV41*03			■				
BV_TCRBV41*04					■	■	■
BV_TCRBV41*05						■	
BV_TCRBV42*01	■	■	■	■	■	■	■

Supplementary Figure S6. Phylogenetic tree of amino acid sequences of bank vole TCRβ V genes retrieved in the present study, bank vole TCRβ V genes automatically extracted from a WGA, and mouse reference TCRβ V sequences. The evolutionary history was inferred using the neighbour-joining method. Bootstrap values greater than 50, based on 1000 replicates, are shown. Evolutionary distances were computed using the JTT matrix-based method and are in the units of the number of amino acid substitutions per site. Orange circles mark the mouse sequences (TRBVXX*01); red circles indicate murine pseudogenes (P) or sequences in an open reading frame (ORF) but with alterations in regulatory elements or splice sites; blue circles mark the bank vole sequences (BV_TRBVXX*01); and green circles denote bank vole TCRβ V genes automatically extracted from a WGA. Red branches mark clades (subgroups) absent from the bank vole WGA, but represented in the genes retrieved in this study. A full list of mouse reference sequences with accession numbers is available in Supplementary Table S1; sequences of bank vole genes identified in this study are available in Supplementary File S1 and S2; and V genes extracted from WGA (GenBank assembly ID: LIPI00000000.1) are available in an online database, Vgenerepertoire.org.

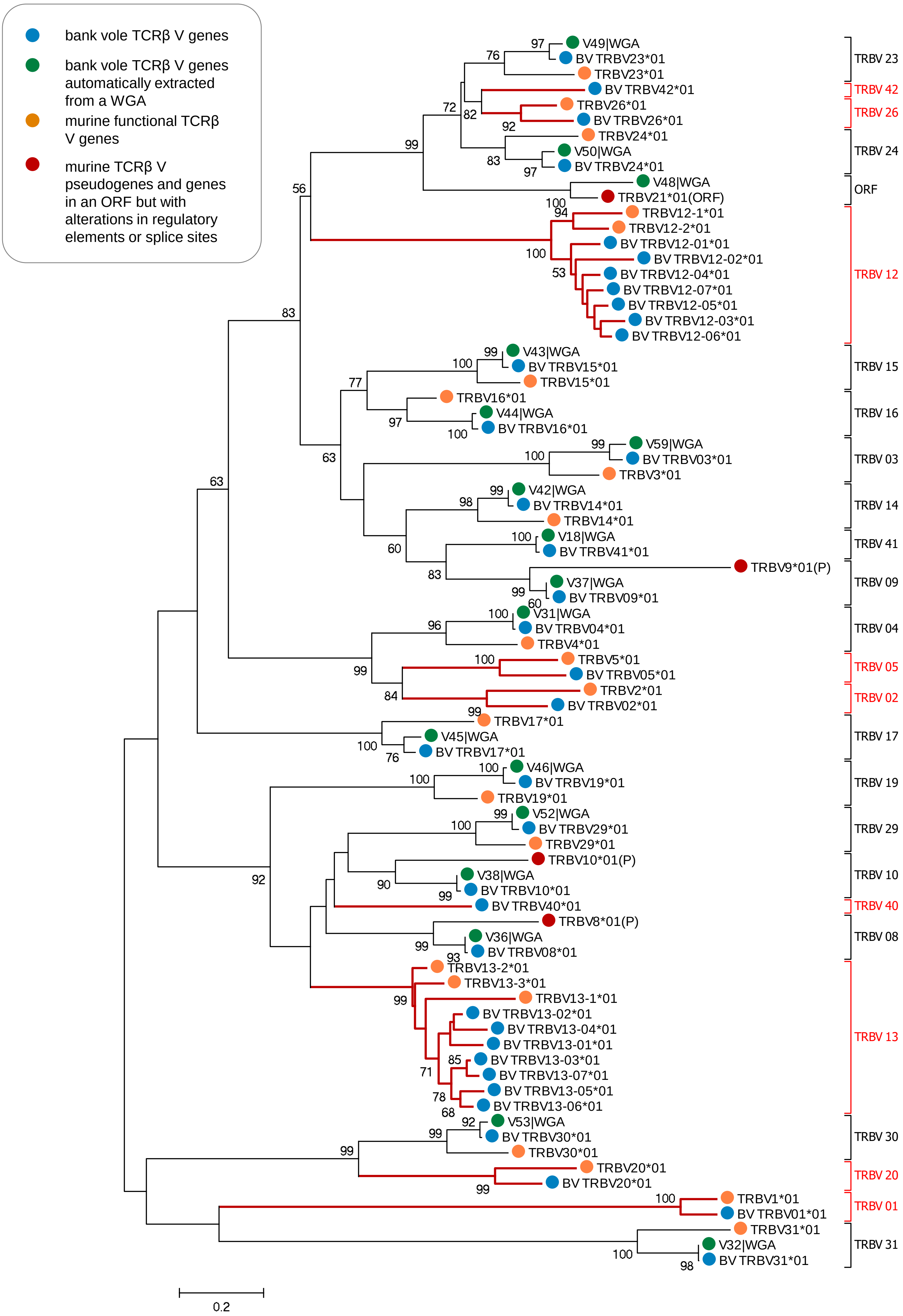

bank vole TCRβ V genes
bank vole TCRβ V genes automatically extracted from a WGA
murine functional TCRβ V genes
murine TCRβ V pseudogenes and genes in an ORF but with alterations in regulatory elements or splice sites
V49|WGA
BV TRBV23*01
TRBV23*01
BV TRBV42*01
TRBV26*01
BV TRBV26*01
TRBV24*01
V50|WGA
BV TRBV24*01
V48|WGA
TRBV21*01(ORF)
TRBV12-1*01
TRBV12-2*01
BV TRBV12-01*01
BV TRBV12-02*01
BV TRBV12-04*01
BV TRBV12-07*01
BV TRBV12-05*01
BV TRBV12-03*01
BV TRBV12-06*01
V43|WGA
BV TRBV15*01
TRBV15*01
TRBV16*01
V44|WGA
BV TRBV16*01
V59|WGA
BV TRBV03*01
TRBV3*01
V42|WGA
BV TRBV14*01
TRBV14*01
V18|WGA
BV TRBV41*01
TRBV9*01(P)
V37|WGA
BV TRBV09*01
V31|WGA
BV TRBV04*01
TRBV4*01
TRBV5*01
BV TRBV05*01
TRBV2*01
BV TRBV02*01
TRBV17*01
V45|WGA
BV TRBV17*01
V46|WGA
BV TRBV19*01
TRBV19*01
V52|WGA
BV TRBV29*01
TRBV29*01
TRBV10*01(P)
V38|WGA
BV TRBV10*01
BV TRBV40*01
TRBV8*01(P)
V36|WGA
BV TRBV08*01
TRBV13-2*01
TRBV13-3*01
TRBV13-1*01
BV TRBV13-02*01
BV TRBV13-04*01
BV TRBV13-01*01
BV TRBV13-03*01
BV TRBV13-07*01
BV TRBV13-05*01
BV TRBV13-06*01
V53|WGA
BV TRBV30*01
TRBV30*01
TRBV20*01
BV TRBV20*01
TRBV1*01
BV TRBV01*01
TRBV31*01
V32|WGA
BV TRBV31*01
TRBV 23
TRBV 42
TRBV 26
TRBV 24
ORF
TRBV 12
TRBV 15
TRBV 16
TRBV 03
TRBV 14
TRBV 41
TRBV 09
TRBV 04
TRBV 05
TRBV 02
TRBV 17
TRBV 19
TRBV 29
TRBV 10
TRBV 40
TRBV 08
TRBV 13
TRBV 30
TRBV 20
TRBV 01
TRBV 31
0.2

Supplementary Figure S7. Differences in the TCRβ V–J segment paring frequencies in all seven individuals (s01-s07). Heatmap of frequencies of the CDR3 variants formed by the given V–J combinations.

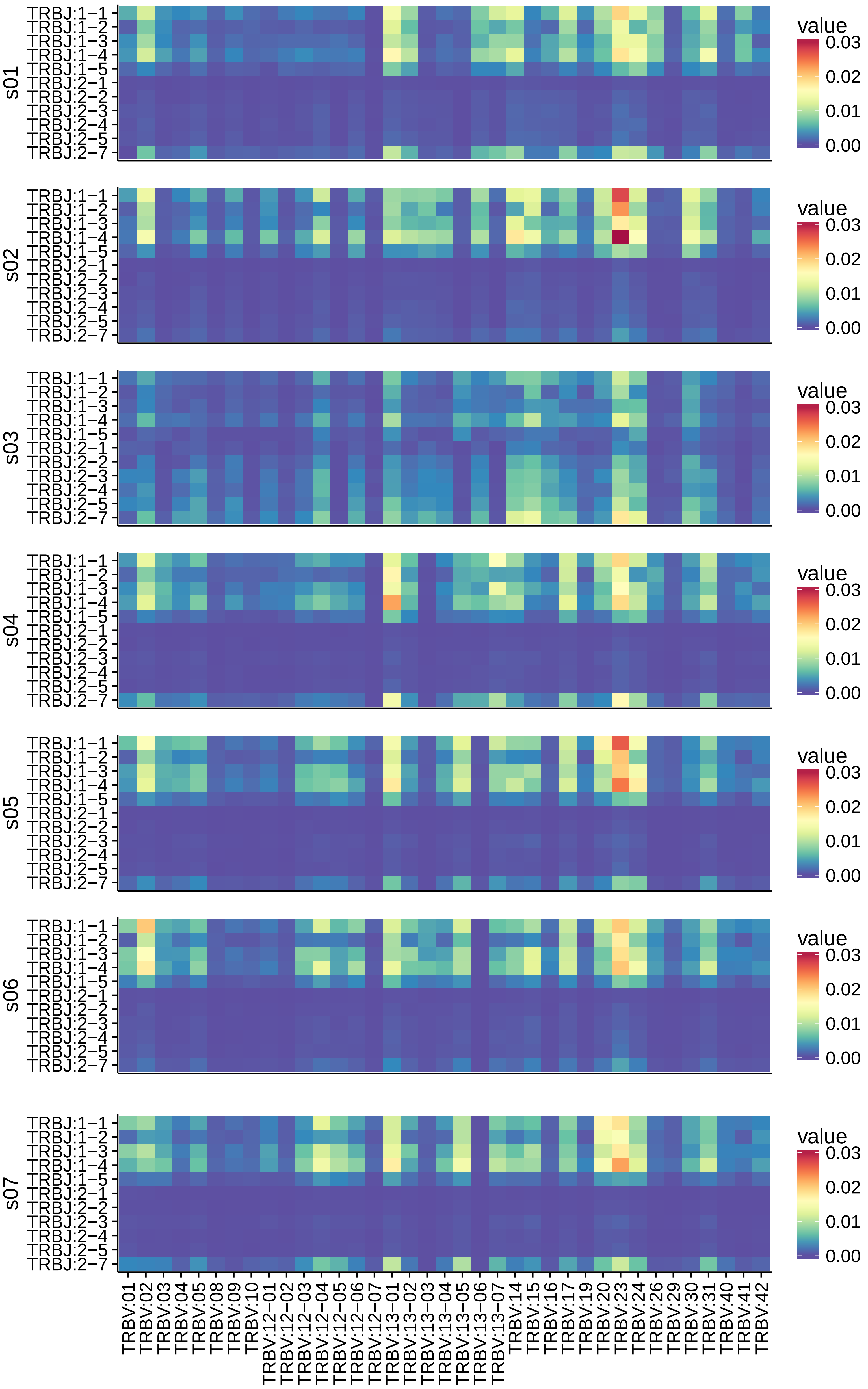

s01
s02
s03
s04
s05
s06
s07
TRBJ:1−1
TRBJ:1−2
TRBJ:1−3
TRBJ:1−4
TRBJ:1−5
TRBJ:2−1
TRBJ:2−2
TRBJ:2−3
TRBJ:2−4
TRBJ:2−5
TRBJ:2−7
value
0.03
0.02
0.01
0.00
TRBV:01
TRBV:02
TRBV:03
TRBV:04
TRBV:05
TRBV:08
TRBV:09
TRBV:10
TRBV:12−01
TRBV:12−02
TRBV:12−03
TRBV:12−04
TRBV:12−05
TRBV:12−06
TRBV:12−07
TRBV:13−01
TRBV:13−02
TRBV:13−03
TRBV:13−04
TRBV:13−05
TRBV:13−06
TRBV:13−07
TRBV:14
TRBV:15
TRBV:16
TRBV:17
TRBV:19
TRBV:20
TRBV:23
TRBV:24
TRBV:26
TRBV:29
TRBV:30
TRBV:31
TRBV:40
TRBV:41
TRBV:42

Supplementary Figure S8. CDR3 *in silico* spectratyping. CDR3 amino acid length distribution with bar stacks colour-coded according to the relative contribution of each TRBJ (a) and TRBV (b) gene.

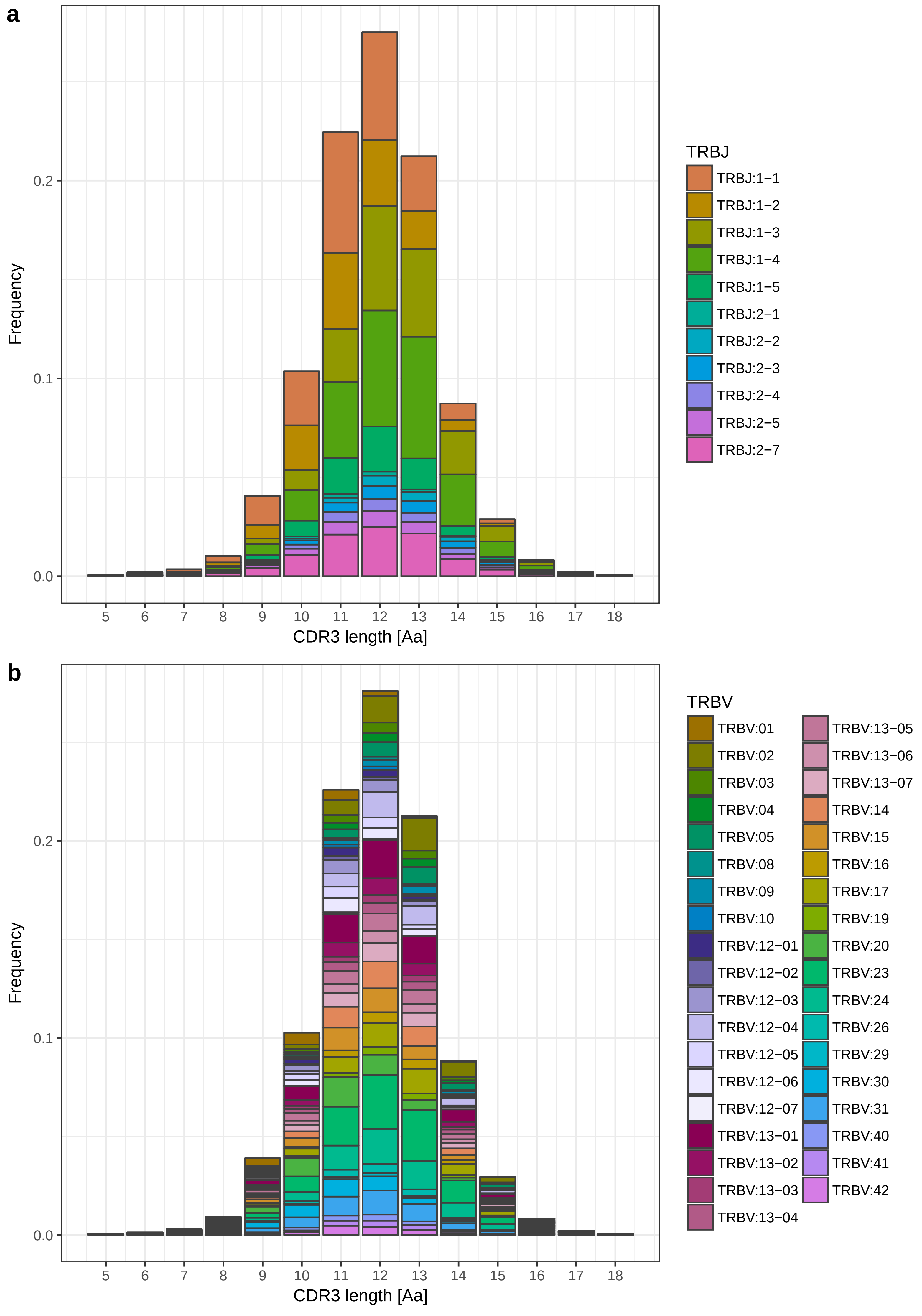
a
Frequency
0.0
0.1
0.2
5
6
7
8
9
10
11
12
13
14
15
16
17
18
CDR3 length [Aa]
TRBJ
TRBJ:1−1
TRBJ:1−2
TRBJ:1−3
TRBJ:1−4
TRBJ:1−5
TRBJ:2−1
TRBJ:2−2
TRBJ:2−3
TRBJ:2−4
TRBJ:2−5
TRBJ:2−7
b
Frequency
0.0
0.1
0.2
5
6
7
8
9
10
11
12
13
14
15
16
17
18
CDR3 length [Aa]
TRBV
TRBV:01
TRBV:02
TRBV:03
TRBV:04
TRBV:05
TRBV:08
TRBV:09
TRBV:10
TRBV:12−01
TRBV:12−02
TRBV:12−03
TRBV:12−04
TRBV:12−05
TRBV:12−06
TRBV:12−07
TRBV:13−01
TRBV:13−02
TRBV:13−03
TRBV:13−04
TRBV:13−05
TRBV:13−06
TRBV:13−07
TRBV:14
TRBV:15
TRBV:16
TRBV:17
TRBV:19
TRBV:20
TRBV:23
TRBV:24
TRBV:26
TRBV:29
TRBV:30
TRBV:31
TRBV:40
TRBV:41
TRBV:42

Supplementary Figure S9. Sharing of the unique CDR3 sequences. Number of unique nucleotide (a) and amino acid (b) TCRβ CDR3 sequences shared among three individuals analysed in the quantitative part of the study.

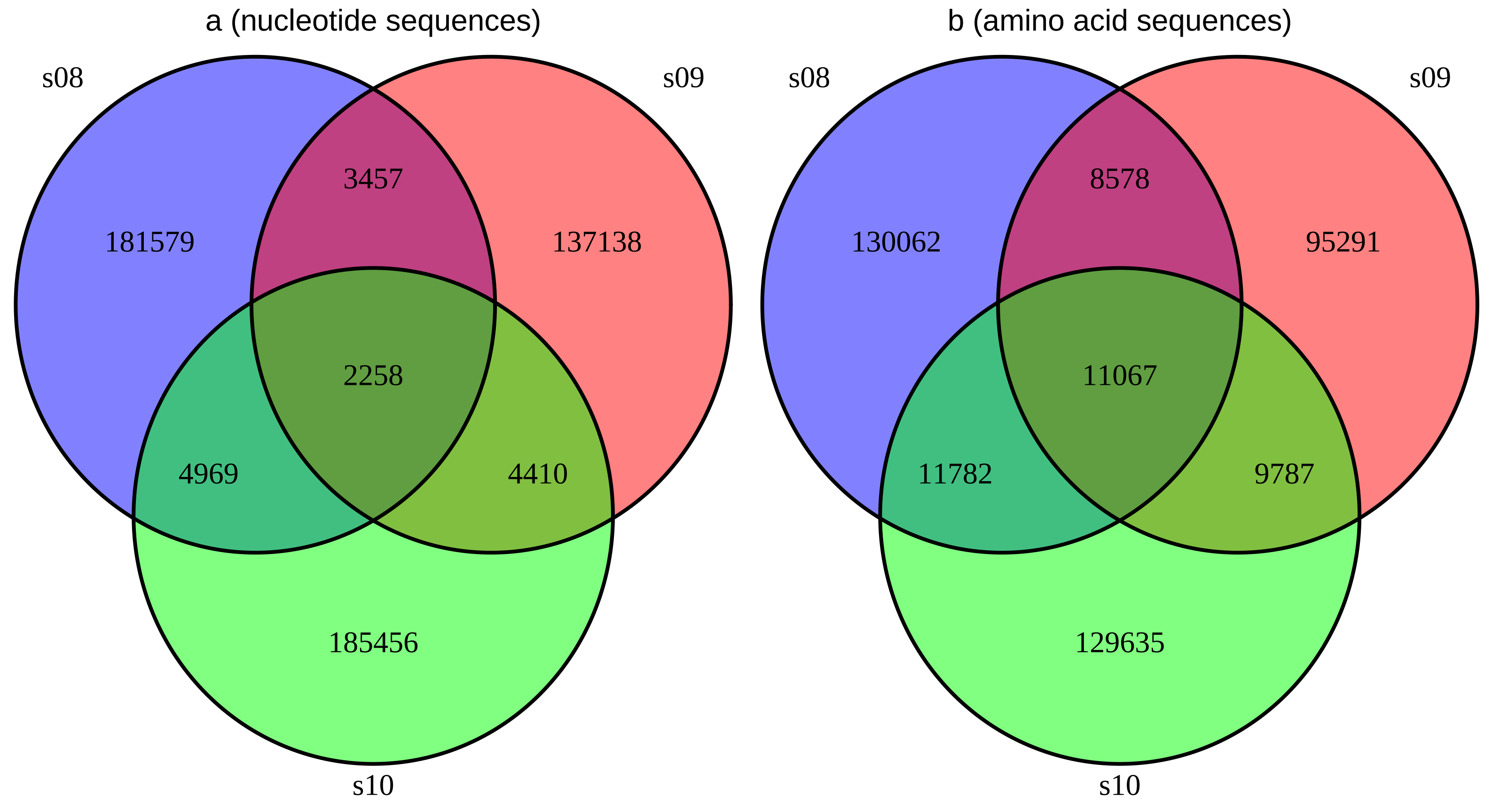
a (nucleotide sequences)
s08
s09
s10
181579
3457
137138
2258
4969
4410
185456
b (amino acid sequences)
s08
s09
s10
130062
8578
95291
11067
11782
9787
129635

Supplementary Figure S10. Rarefaction curve. For each replicated amplicon (A–D) in three individuals (s08–s10), reads were subsampled at depths 0.25, 0.5, 0.75, 1, 1.5, 2, 2.5, 3, 3.5, and 4 mln and a number of retrieved, UMI-corrected, unique CDR3 variants was plotted.

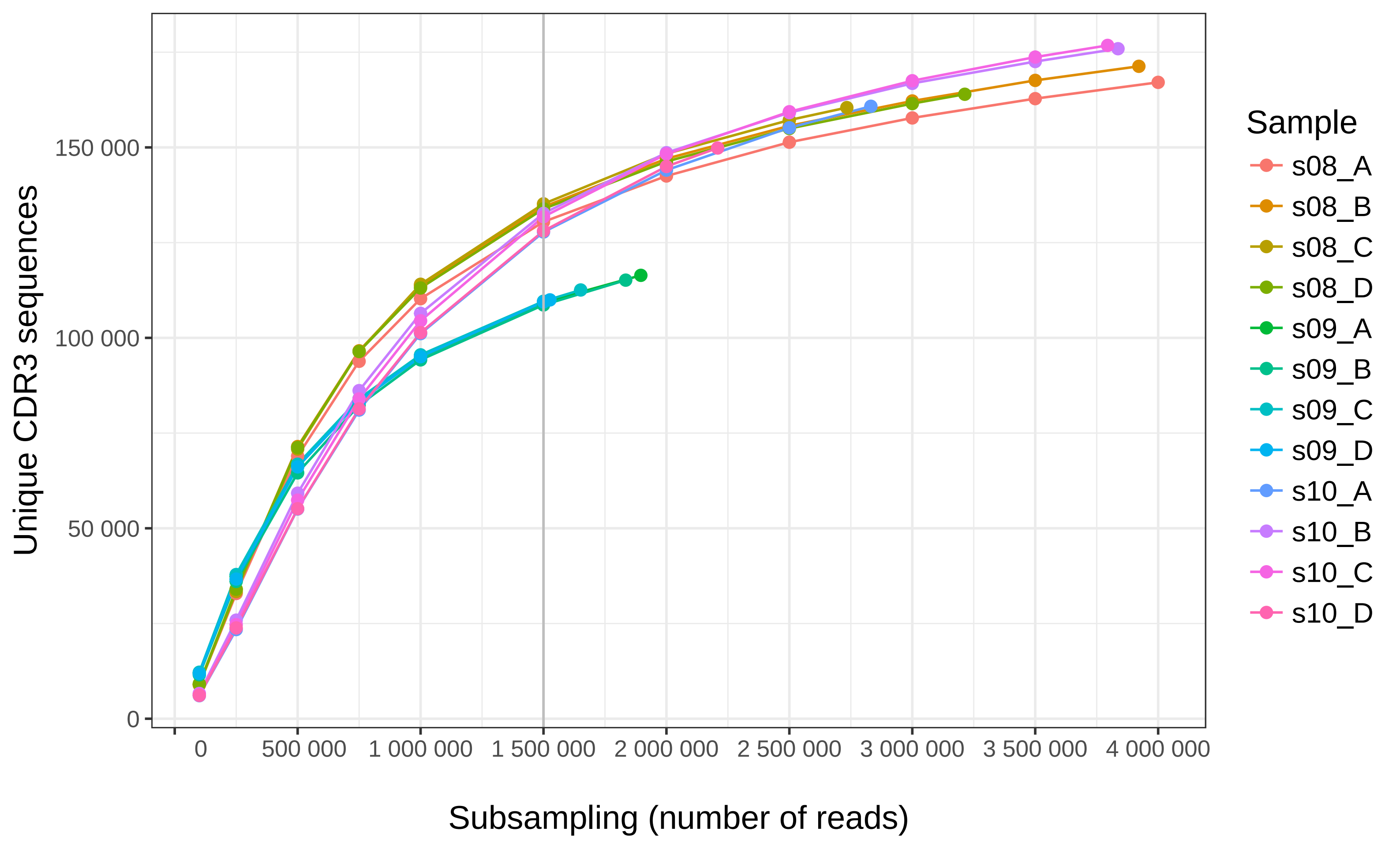
Unique CDR3 sequences
150 000
100 000
50 000
0
0
500 000
1 000 000
1 500 000
2 000 000
2 500 000
3 000 000
3 500 000
4 000 000
Subsampling (number of reads)
Sample
s08_A
s08_B
s08_C
s08_D
s09_A
s09_B
s09_C
s09_D
s10_A
s10_B
s10_C
s10_D

Supplementary Tables

Supplementary Table S1. List of mouse reference sequences with accession numbers from IMGT® database.

Segment name	Gene and allele name	IMGT/LIGM-DB accession number	*Mus musculus* strain	Functionality	Start and end positions in the IMGT/LIGM-DB accession number	Number of nucleotides in the IMGT/LIGM-DB accession number	Start codon
V (variable)	TRBV1*01	AE000663	BALB/c	functional	13939--14225	287 nt	1
	TRBV2*01	AE000663	BALB/c	functional	169947--170233	287 nt	1
	TRBV3*01	AE000663	BALB/c	functional	170773--171062	290 nt	1
	TRBV4*01	X56725	C57BL/6	functional	174--460	287 nt	1
	TRBV5*01	AE000663	BALB/c	functional	184762--185045	284 nt	1
	TRBV8*01	AE000663	BALB/c	pseudogene	207058--207344	287 nt	1
	TRBV9*01	X16693	BALB/c	pseudogene	1--290	290 nt	1
	TRBV10*01	X16694	BALB/c	pseudogene	1--289	289 nt	1
	TRBV12-1*01	M15614	C57BL/6	functional	229--513	285 nt	1
	TRBV12-2*01	M15613	C57BL/6	functional	235--519	285 nt	1
	TRBV13-1*01	M15618	C57BL/6	functional	158--443	286 nt	1
	TRBV13-2*01	M15617	C57BL/6	functional	160--445	286 nt	1
	TRBV13-3*01	M15616	C57BL/6	functional	164--449	286 nt	1
	TRBV14*01	AE000664	BALB/c	functional	7006--7294	289 nt	1
	TRBV15*01	AE000664	BALB/c	functional	13044--13333	290 nt	1
	TRBV16*01	L29434	C57BL/6	functional	844--1133	290 nt	1
	TRBV17*01	AE000664	BALB/c	functional	34935--35221	287 nt	1
	TRBV19*01	AE000664	BALB/c	functional	50425--50710	286 nt	1
	TRBV20*01	AE000664	BALB/c	functional	60348--60640	293 nt	1
	TRBV21*01	X16691	BALB/c	ORF	1--290	290 nt	1
	TRBV23*01	AE000664	BALB/c	functional	87900--88192	290 nt	1
	TRBV24*01	M61184	PWK	functional	178--467	290 nt	1
	TRBV26*01	K02548	B10-A	functional	2--291	290 nt	1
	TRBV29*01	AE000664	BALB/c	functional	143112--143398	287 nt	1
	TRBV30*01	X16695	BALB/c	functional	1--290	290 nt	1
	TRBV31*01	X03277	BALB/c	functional	427--710	284 nt	1

J (joining)	TRBJ1-1*01	X01018	B10.A	functional	73..120	48 nt	3
	TRBJ1-2*01	X01018	B10.A	functional	209..256	48 nt	3
	TRBJ1-3*01	X01018	B10.A	functional	549..598	50 nt	2
	TRBJ1-4*01	X01018	B10.A	functional	1036..1086	51 nt	3
	TRBJ1-5*01	AE000665	BALB/c	functional	155176..155225	50 nt	2
	TRBJ1-6*01	X01018	B10.A	ORF	1778..1830	53 nt	2
	TRBJ2-1*01	K02802	B10.A	functional	405..454	50 nt	2
	TRBJ2-2*01	K02802	B10.A	functional	608..658	51 nt	3
	TRBJ2-3*01	K02802	B10.A	functional	874..922	49 nt	1
	TRBJ2-4*01	K02802	B10.A	functional	1013..1061	49 nt	1
	TRBJ2-5*01	K02802	B10.A	functional	1104..1152	49 nt	1
	TRBJ2-7*01	K02802	B10.A	functional	1460..1506	47 nt	2

Supplementary Table S2. Quantitative **analysis of the bank vole TCRβ repertoire.**

Sample	Raw reads	Pre-processed reads	Subsampled reads	Total CDR3s	Filtered CDR3s	UMI-corrected, unique CDR3 [nt]	Translated, unique CDR3s [aa]
**s08_A**	4 348 883	3 715 190	1 500 000	1 444 126	1 027 504	135 523	116 998
**s08_B**	3 921 528	3 328 499	1 500 000	1 440 381	1 061 826	140 113	120 997
**s08_C**	2 733 740	2 325 749	1 500 000	1 436 925	1 051 260	140 900	121 867
**s08_D**	3 213 527	2 721 862	1 500 000	1 441 385	1 064 853	139 229	120 339
**s09_A**	1 895 944	1 604 826	1 500 000	1 459 126	1 125 212	112 995	98 131
**s09_B**	1 834 330	1 562 509	1 500 000	1 457 522	1 086 560	112 430	97 443
**s09_C**	1 651 161	1 407 176	1 407 176	1 368 393	1 058 349	111 239	96 792
**s09_D**	1 526 104	1 292 617	1 292 617	1 256 157	960 428	108 487	94 479
**s10_A**	2 831 557	2 518 003	1 500 000	1 341 979	869 573	132 567	112 848
**s10_B**	3 836 747	3 429 632	1 500 000	1 372 538	921 497	137 088	116 557
**s10_C**	3 794 421	3 389 887	1 500 000	1 374 509	901 547	136 551	116 124
**s10_D**	2 208 357	1 942 147	1 500 000	1 347 847	883 212	133 600	113 825
**MEAN**	2 816 358	2 436 508	1 474 983	1 395 074	1 000 985	128 394	110 533
**SD**	954 650	841 339	60 631	59 405	84 680	12 353	10 130

Supplementary Table S3. Lower bound of the bank vole TCR repertoire size estimated with Chao2 estimator.

**Individual**	**q_1**	**q_2**	**q_3**	**q_4**	**S_{obs}**	**Chao2**	**Sobs/Chao2**
**S08**	45 115	23 649	30 126	93 373	192 263	224 538	85.6%
	23.5%	12.3%	15.7%	48.6%			
**s09**	30 183	15 862	21 600	79 618	147 263	168 801	87.2%
	20.5%	10.8%	14.7%	54.1%			
**s10**	49 978	29 203	40 586	77 416	197 183	229 258	86.0%
	25.3%	14.8%	20.6%	39.3%			
**mean**	**41 759**	**22 905**	**30 771**	**83 469**	**178 903**	**207 532**	**86.3%**
mean%	23.1%	12.6%	17.0%	47.3%			

Legend:
q_1 - number of CDR3 sequences present in only one replicate (amplicon)
q_2 - number of CDR3 sequences present in two replicates (amplicons)
q_3 - number of CDR3 sequences present in three replicates (amplicons)
q_4 - number of CDR3 sequences present in all four replicates (amplicons)
S_{obs} - the total number of CDR3 unique sequences observed across all replicates for a given individual
Chao2 - non-parametric, incidence-based Chao2 estimator (Chao, 1987)

Supplementary Table S4. Primers and adaptors used in 5' RACE (5'-3' orientation).

Stage	Name	Usage	Description	Sequence (5'→ 3')	Source
First strand cDNA synthesis	PlugOligo-1	QL	5' template switch adapter (available with the kit)	AAGCAGTGGTATCAACGCAGAGTACGCGGG	A
	SmartNNNNa	QN	5' template switch adapter with UMI (sequenced by Integrated DNA Technologies®)	AAGCAGUGGTAUCAACGCAGAGUNNNNUNNNNUNNNNUCTTrGrGrGrG	B
	MyglTCRb_1	QL, QN	Primer for cDNA synthesis, bank vole TCRβ mRNA constant region	TGATCTCTGCTTCTGATG	C
First PCR amplification	Smart20	QL	Step – out primer, anneals to the Switch_oligo	CACTCTATCCGACAAGCAGTGGTATCAACGCAG	D
	MyglTCRb_2	QL	Nested primer for bank vole TCRβ constant region	GATGGCTCAAACAGGGTGACC	C
	Smart20-mod	QN	Step-out primer carrying fragment of Illumina sequencing primer, anneals to SmartNNNNa	TCGTCGGCAGCGTCAGATGTGTATAAGAGACAG(N$_{0-6}$)GTAAGCAGTGGTATCAACGCAG	E
	MyglTCRb_3NN	QN	Nested primer for bank vole TCRβ constant region carrying a fragment of Illumina sequencing primer	GTCTCGTGGGCTCGGAGATGTGTATAAGAGACAG(N$_{0-4}$)GGACTCACCTTGCTCAGATCCT	E
Second PCR amplification	Step_1	QL	Step – out primer, anneals to the Smart 20	CACTCTATCCGACAAGCAGT	D
	MyglTCRb_3	QL	Nested primer for bank vole TCRβ constant region	GGACTCACCTTGCTCAGATCCT	C
	P5_50X	QN	Primer carrying Illumina P5 adaptor sequence and i5 index, anneals to the Smart20-mod	AATGATACGGCGACCACCGAGATCTACACXXXXXXTCGTCGGCAGCGTC	E
	P7-70X	QN	Primer carrying Illumina P7 adaptor sequence and i7 index, anneals to the MyglTCRb_3NN	CAAGCAGAAGACGGCATACGAGATXXXXXXGTCTCGTGGGCTCGG	E

Legend:
U - deoxyuracil
rG - riboguanine
(N$_{0-x}$) – random nucleotides introduced to diversify samples and promote better cluster identification on Illumina® instrument
XXXXXX – sample barcode corresponding to Nextera Index Kit indices (Illumina®)

Primer/adaptor sequence sources:
A: Sequence and name correspond to Mint-2 cDNA synthesis kit component, Cat # SK005 (Evrogen, Moscow, Russia)
B: Sequence and name correspond to Shugay *et al.* 2014
C: Primers designed in this study
D: Sequences and names correspond to Mamedov *et al.* 2013
E: Primers designed in this study with fragments of published Illumina® sequencing primers and/or adaptors

Usage (type of analysis):
QL: Qualitative description of the bank vole TCRβ repertoire - V and J segments identification and naming, V-J segment usage, CDR3 length distribution
QN: Quantitative analysis of the bank vole TCRβ repertoire - repertoire size estimation, private vs. public repertoire

Supplementary Table S5. Experimental animals.

Individual	Sex	Age at death [days]	Analysis type
			Qualitative/Quantitative
S01	F	120	QL
S02	F	118	QL
S03	M	118	QL
S04	M	117	QL
S05	M	117	QL
S06	M	116	QL
S07	M	112	QL
S08	F	130	QN
S09	F	126	QN
S10	F	123	QN

Supplementary Table S6. Qualitative **description of the bank vole TCRβ repertoire.**

Individual	Raw reads	Pre-processed reads (max 2 mln)	Total CDR3s	Filtered CDR3s	De-replicated (unique) CDR3s	Singletons CDR3s	Translated CDR3s
S01	2 874 831	1 735 702	1 050 290	899 825	105 628	72 032	85 541
S02	2 958 342	1 830 763	819 897	730 795	81 267	54 002	65 655
S03	2 562 446	1 584 306	924 654	694 139	81 887	47 850	67 526
S04	3 252 316	1 946 828	1 050 847	897 039	85 606	58 657	68 344
S05	2 756 794	1 718 934	1 040 778	912 973	123 218	74 113	97 205
S06	3 104 526	1 849 465	1 168 484	1 040 209	119 892	78 807	94 989
S07	2 850 275	1 767 252	848 956	729 903	85 437	54 758	69 480
**Mean**	**2 908 504**	**1 776 179**	**986 272**	**843 555**	**97 562**	**62 888**	**78 391**
**SD**	**209 220**	**106 423**	**116 357**	**118 004**	**17 000**	**11 033**	**12 768**
**MIN**	2 562 446	1 584 306	819 897	694 139	81 267	47 850	65 655
**MAX**	3 252 316	1 946 828	1 168 484	1 040 209	123 218	78 807	97 205

Supplementary Table S7. Comparison of murine TCRβ repertoire basic statistics obtained with AmpliCDR3 and MIGEC/MiXCR.
Basic data and diversity metrics for the CDR3 repertoires from PBMC of eight young (3-month-old) and eight old (23-month-old) C57BL/6J mice. The analysis was performed for a normalized dataset, down-sampled to 15 000 randomly chosen UMIs. HTS sequencing data used for this comparison was originally generated (NCBI BioProject: PRJNA341443) and analysed by Izraelson *et al.*, 2018. Results shown for MIGEC (Shugay *et al.*, 2014)/MiXCR (Bolotin *et al.*, 2015) analysis were taken from Supplementary Table S1 of Izraelson *et al.*, 2018.

**#Sample_ID**	**Observed diversity**		**Chao1**		**Shannon-Wiener**	
	**MIGEC**	**AmpliCDR3**	**MIGEC**	**AmpliCDR3**	**MIGEC**	**AmpliCDR3**
old_1	5 303	5 299	12 561	12 981	1 403	1 355
old_2	4 341	4 385	11 168	11 170	598	619
old_3	3 050	3 135	12 342	14 217	46	48
old_4	5 984	5 982	13 671	13 886	2 407	2 364
old_6	6 279	6 419	13 929	14 433	1 934	2 028
old_7	2 402	2 470	85 84	9 657	50	50
old_8	4 322	4 537	10 334	10 652	761	865
old_9	5 724	5 849	13 690	14 638	2 002	2 077
young _1	10 792	10 977	82 754	87 961	5 720	5 899
young_2	12 115	12 197	116 403	126 547	8 879	9 037
young_3	13 467	13 502	131 194	132 922	12 292	12 304
young_4	12 891	13 000	123 146	128 708	11 114	11 309
young_5	12 236	12 315	128 455	137 610	9 044	9 097
young_6	11 033	11 226	108 291	108 069	6 559	6 894
young_7	13 046	13 073	142 580	138 039	11 079	11 155
young_8	12 356	12 346	108 322	108 126	8 968	8 987

References

Bolotin DA, Poslavsky S, Mitrophanov I, Shugay M, Mamedov IZ, Putintseva E V, *et al.* (2015). MiXCR: software for comprehensive adaptive immunity profiling. *Nat Methods* **12**: 380–381.

Chao A (1987). Estimating the population size for capture-recapture data with unequal catchability. *Biometrics* **43**: 783–791.

Izraelson M, Nakonechnaya TO, Moltedo B, Egorov ES, Kasatskaya SA, Putintseva E V., *et al.* (2018). Comparative analysis of murine T-cell receptor repertoires. *Immunology* **153**: 133–144.

Mamedov IZ, Britanova O V, Zvyagin I V, Turchaninova M a, Bolotin D a, Putintseva E V, *et al.* (2013). Preparing unbiased T-cell receptor and antibody cDNA libraries for the deep next generation sequencing profiling. *Front Immunol* **4**: 456.

Shugay M, Britanova O V, Merzlyak EM, Turchaninova M a, Mamedov IZ, Tuganbaev TR, *et al.* (2014). Towards error-free profiling of immune repertoires. *Nat Methods* **11**: 653–5.

Supplementary Files

Supplementary File S1.

Protocol:
T-cell receptor cDNA library preparation by 5'RACE for deep HTS profiling

The protocol was based on work of Mamedov et al. (2013)[1] and *User manual* of Mint-2 cDNA synthesis kit (Evrogen). It was further modified to describe both ligation and primer extension method of adaptor addition.

- The sequences of the nested primers complementary to 3' ending of the constant region of the TCR will differ depending on I) species; II) receptor chain of interest. This protocol uses primers for the bank vole TCRβ chain, which should be substituted with appropriate primers when other species/chain are analysed.

- The protocol will have to be adjusted if another 5'RACE kit provider is chosen.

- A necessary step of amplicon library preparation for HTS is the addition of platform-specific sequencing adapters. Here, we present steps for either I) ligation, or II) two-step PCR-based primer extension method (with modified primers containing partial Illumina® adaptor sequences and indices). The latter method requires lower amounts of starting RNA and was proven to be equally accurate, but more efficient and less time-consuming[2] than ligation. However, especially with pooling of multiple samples, it requires synthesis of dozens of additional primers.

- The protocol is designed for the Illumina® paired-end sequencing. For better cluster differentiation during sequencing, spike the library with 15-20% of PhiX control library. 2×300bp sequencing will cover the entire Variable domain: whole V, D, J segments and the CDR3. It allows *de novo* description of the V and J segments and all qualitative and quantitative analysis. 2×150bp sequencing will cover entire J segment and CDR3, but only partially cover V segment. It is sufficient for detailed CDR3 analysis, but detailed characteristics of e.g. V-J usage can be compromised without thorough reference information for V segments.

References:

1. Mamedov, I. Z. *et al.* Preparing unbiased T-cell receptor and antibody cDNA libraries for the deep next generation sequencing profiling. *Front. Immunol.* **4,** 456 (2013).

2. Menzel, U. *et al.* Comprehensive Evaluation and Optimization of Amplicon Library Preparation Methods for High-Throughput Antibody Sequencing. *PLoS One* **9,** e96727 (2014).

3. Shugay, M. *et al.* Towards error-free profiling of immune repertoires. *Nat. Methods* **11,** 653–5 (2014).

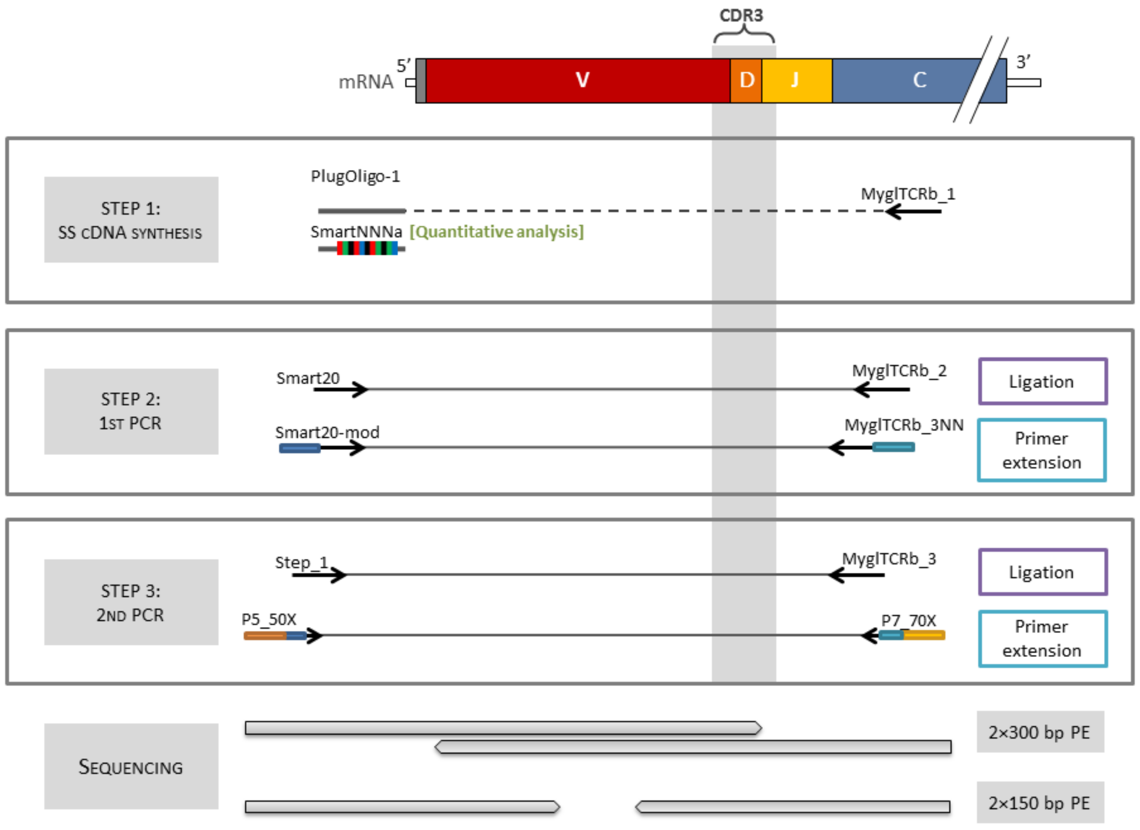

Figure 1. Simplified overview of the library preparation protocol from RNA with 5'RACE for deep HTS profiling. For details on primers and adaptors see Supplementary Table S4. Suggested sequencing method: Illumina, paired-end (PE) sequencing.

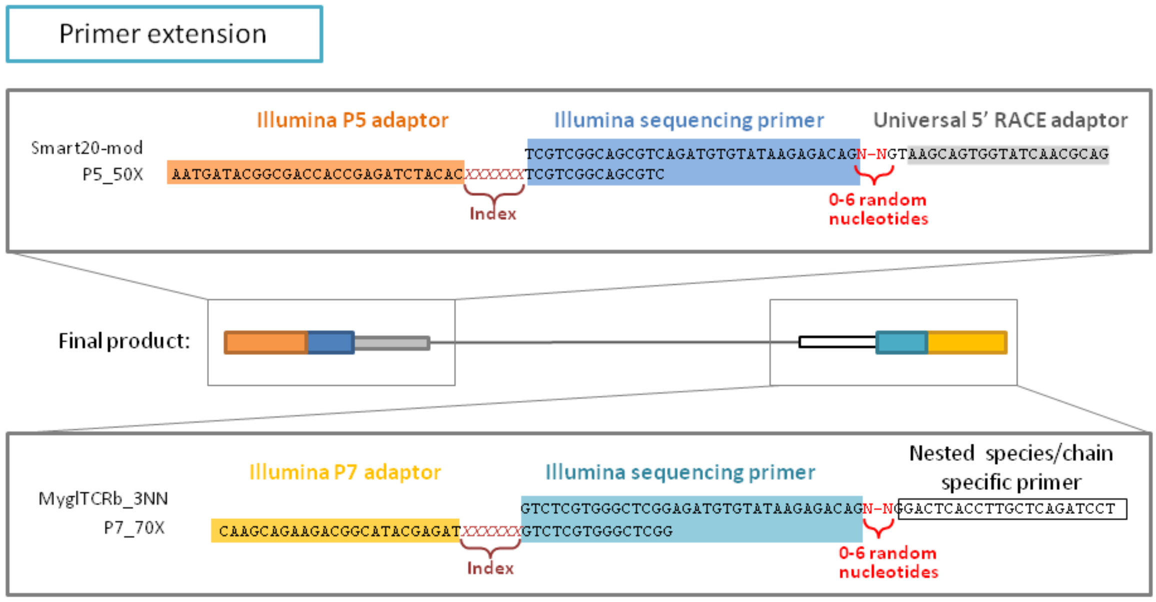

Figure 2. Two-step primer extension method for library preparation. Generation of full, functional Illumina adaptors through primer-extension. Random nucleotides (0-6) are added to introduce diversity at the beginning of the read and promote better cluster identification on Illumina instruments. All primer/adaptor sequences are in 5'→3' direction.

Materials and reagents

- For primer/adaptor sequences see **Supplementary Table S4** of the original article.
- Quantitative analysis with UMIs: it is crucial to synthesize **SmartNNNa**[3]**.** adaptors in a high-quality facility, to assure 'randomness' of unique molecular identifiers (UMIs). Several provides may perform satisfactory, we can recommend Integrated DNA Technologies (IDT).
- Reagents needed only for quantitative analysis are marked with **QN** symbol.
- If the primer extension method is chosen for addition of platform-specific sequencing adaptors certain primer modifications are necessary.

- **Mint-2 cDNA synthesis kit, Evrogen** (Cat# SK005) – or else, with template-switch activity. This kit includes polymerase for subsequent PCRs; if a different kit is used - suitable, high-fidelity polymerase has to be also purchased. The Evrogen kit consists of:
  - First-strand buffer
  - DTT (20mM)
  - dNTP mix (10mM each)
  - Mint reverse transcriptase
  - IP-solution
  - 50X Encyclo polymerase mix
  - 10X Encyclo buffer
  - Set of various adapters for cDNA synthesis

- **PCR purification Kit** (e.g. QIAquick, QIAGEN)/**Agencourt AMPure XP beads [QN]**

- **Gel Extraction Kit** (e.g. QIAquick, QIAGEN)

- **RNase, DNase free water; RNase, DNase free plasticware**

- **USER™ enzyme, New England Biolabs** (M5505S) **[QN]**

- **Primers and adaptors:**

  STEP 1 (SS cDNA SYNTHESIS)
  - **5' adapter:** PlugOligo-1 (in Mint-2 cDNA synthesis kit, Evrogen) **OR SmartNNNNa**[3] **[QN]**
  - **Target-specific primer for cDNA synthesis** - complementary to 3' ending of the constant region of the TCR receptor chain of interest (here: bank vole TCRβ, *MyglTCRb_1*).

  STEP 2 (1ST PCR)
  - **First step-out primer** Smart20[1].
    Primer has to be modified if primer-extension method is chosen instead of ligation (see: *Smart20-mod*).
  - **First target-specific PCR primer**, complementary to 3' ending of the constant region of the TCR receptor chain of interest (here: *MyglTCRb_2*).
    Primer has to be modified if primer-extension method is chosen instead of ligation (see: *MyglTCRb_3NN*).

  STEP 3 (2ND PCR)
  - **Second step-out primer** Step_1[1].
    Primer has to be modified if primer-extension method is chosen instead of ligation. For an example see: *P5-50X.*
  - **Second target-specific PCR primer**, complementary to 3' ending of the constant region of the TCR receptor chain of interest (here: *MyglTCRb_3*).
    Primer has to be modified if primer-extension method is chosen instead of ligation. For an example see: *P7-70X.*

Procedure

A. STEP 1: cDNA SYNTHESIS AND TEMPLATE SWITCH

- The following procedure is for up to 2µg of total RNA/up to 1µg of purified mRNA per 10 µl of final reaction volume. The RNA extraction protocol is not included herein, we recommend e.g., RNazol-based extraction. In some situations, use of purified mRNA may be preferred.
- Reverse transcriptases differ in their 5'-template switching activity, therefore should be chosen with care. We successfully used suggested Mint-2 cDNA synthesis kit, Evrogen.
- Note change in adaptor and additional step for quantitative analysis with UMIs.

1. Prepare 2 separate reaction tubes for two mixes - final volume will be **10 µl**.

2. MIX1 (final volume 4µl):

- RNA	1-3 µl (up to 2µg of total RNA or up to 1µg of polyA RNA in final volume)
- cDNA synthesis primer (*MyglTCRb_1*)	1µl from 10µM stock (1 µM in final volume)
- H_2O, RNAse free	to 4 µl

3. Place a tube with MIX1 into a thermal cycler and incubate for **4 min at 70°C**, and then **2 min at 42°C** (annealing synthesis primers).

4. (Meanwhile) prepare second mix (final volume 6µl):
   MIX2:

- First strand buffer (Evrogen, 5x)	2 µl
- DTT (20uM)	1 µl
- 5'-template switch adapter (15uM) PlugOligo-1 Evrogen **OR SmartNNNNa [QN]**	1 µl
- dNTP solution (10mM each)	1 µl
- Mint Reverese Transcriptase (10x)	1 µl

5. Add MIX2 to MIX1, mix by pipetting, incubate at **42°C for 40-60 min**. Do not remove the reaction tube from the thermal cycler except for the time necessary to add MIX2.

6. Add 5ul of IP solution (MINT reverse transcriptase, Evrogen) and incubate for additional **1h at 42°C.** Do not remove the reaction tube from the thermal cycler except for the time necessary to add IP-solution.

7. After incubation, place the tubes on ice to terminate the first-strand cDNA synthesis.

8. Add 1 ul of Uracyl DNA glycosylase – **USER™ enzyme** – 5U, incubate **1h at 37°C**. **[QN only]**

9. Product can be stored in +4°C for several hours, or at -20°C for up to one month. However, prompt proceeding to the next step is advised.

B. STEP 2: First PCR

- Polymerase with high fidelity and processivity should be used for amplification. Hotstart Encyclo polymerase (Evrogen) included in Mint-2 cDNA synthesis kit has 5'>3' DNA polymerase activity with high processivity and proofreading 3'>5' exonuclease activity.
- If the first-strand cDNA samples were stored at -20°C, pre-heat them at 65°C for 1 min, then mix by gently flicking the tubes before taking aliquots.
- The number of PCR cycles and the volume of template in the PCRs will depend on the staring RNA concentration. For optimization see Mamedov et al. (2013).
- Note change in primers if primer-extension method was chosen for addition of sequencing platform adaptors.

1. In a sterile tube mix the following (final volume: 25 µl):
   - First strand cDNA — 1-2.5 µl
   - dNTP (10mM each) — 0.4 µl (final concentration: 0.16 mM each)
   - F primer Smart20 (10uM) **OR** *Smart20-mod* — 1.25 µl (final concentration: 0.5 µM)
   - R primer (*MyglTCRb_2*, 10 uM) **OR** *MyglTCRb_3NN* — 1.25 µl (final concentration: 0.5 µM)
   - Polymerase (Encyclo, 50x) — 0.5 µl
   - Buffer (10x Encyclo) — 2.5 µl
   - H_2O — up to 25 µl

2. PCR conditions (18-23 cycles):
   Initial denaturation: 95°C for 1 min,
   95°C for 20 s
   65°C for 20 s
   72°C for 50 s
   Final elongation: 2 min

3. PCR products should be purified (e.g. using QIAquick PCR purification Kit, QIAGEN), especially if a pause is needed. This step is important since it removes the residual enzyme activities of proofreading polymerases that can damage the obtained PCR library. If **quantitative protocol** with UMIs (SmartNNN 5' adapter) is implemented we **strongly recommend** to purify the 1st PCR product with **Agencourt AMPure XP beads** with 1:0.6 ratio of DNA:beads, to remove short, nonspecific products and primer/adaptor-dimers.

4. Purified first PCR product can be stored at -20°C for a month.

C. STEP 3: SECOND PCR

- The number of PCR cycles and the volume of template in the PCRs will depend on the staring RNA concentration. For optimization see Mamedov et al. (2013).
- Note change in primers if primer-extension method was chosen for addition of sequencing platform adaptors.

1. In a sterile tube mix the following (final volume: 25 µl):

- First PCR product (purified)	1-5 µl
- dNTP (10mM each)	0.4 µl (final concentration: 0.16 mM each)
- F primer Step_1 (10uM) **OR** *P5-50X*	3.75 µl (final concentration: 1.5 µM)
- R primer (*MyglTCRb_3*, 10 uM)	3.75 µl (final concentration: 1.5 µM)
- **OR** *P7-70X*	
- Polymerase (Encyclo, 50x)	0.5 µl
- Buffer (10x Encyclo)	2.5 µl
- H_2O	up to 25 µl

2. PCR conditions (9-14 cycles):
   Initial denaturation: 95°C for 1 min,
   95°C for 20 s
   65°C for 20 s
   72°C for 50 s
   Final elongation: 5 min

3. Although purification of the product with either QIAquick PCR purification Kit or Agencourt AMPure XP beads is possible, we **strongly recommend** to run the 2nd PCR product on agarose gel and excise bands of desired size (~600 bp), using e.g. QIAquick Gel Extraction Kit (QIAGEN). This step should be carried out as soon as possible after the PCR, to prevent residual enzyme activities, that can damage the obtained PCR library.

4. Libraries can be stored for weeks in -20°C.

D. SEQUENCING

- If ligation was chosen for addition of sequencing platform adaptors, these **must be ligated to the product of the STEP 3** (2nd PCR) with an appropriate kit (e.g. NEBNext® DNA Library Prep Master Mix Set for Illumina®, NEB).

- If primer-extension method was chosen for addition of sequencing platform adaptors, the purified products of the STEP 3 (2nd PCR) are ready for paired-end sequencing.

Author: Magdalena Migalska
Evolutionary Biology Group
Faculty of Biology; Adam Mickiewicz University
ul. Umultowska 89, 61-614 Poznan, Poland;
e-mail: magdalena.migalska@amu.edu.pl

Supplementary File S2.

```
>BV_TRBJ1-1*01
GGTAAAGGAACCAGGCTCACAGTG
>BV_TRBJ1-2*01
GGCTCAGGGACCAGGCTTTTGGTA
>BV_TRBJ1-2*02
GGCTCAGGGACCAAGCTTTTGGTA
>BV_TRBJ1-2*03
GGCTCAGGGACCATGCTTTTGGTA
>BV_TRBJ1-3*01
GGAGAAGGAAGTCGGCTCACTGTT
>BV_TRBJ1-3*02
GGAGAAGGAAGCCAGCTCACTGTT
>BV_TRBJ1-3*03
GGAGAAGGAAGCCGGCTCACTGTT
>BV_TRBJ1-4*01
GGTCATGGAACCAAGCTGTCTGTC
>BV_TRBJ1-5*01
GGAGAGGGGACTCGACTGTCTGTT
>BV_TRBJ2-1*01
GGACCAGGCACGCGACTCACGGTG
>BV_TRBJ2-2*01
GGAGAAGGCTCCAAGTTGATAGTG
>BV_TRBJ2-3*01
GGCCCAGGAACCAGACTGACCGTG
>BV_TRBJ2-3*02
GGTCCAGGAACCAGACTGACCGTG
>BV_TRBJ2-4*01
GGTGCAGGCACCCGATTGACAGTC
>BV_TRBJ2-4*02
GGTGCAGGCACCCGATTGACAGTT
>BV_TRBJ2-5*01
GGGCCAGGCACGCGTCTCCTCGTG
>BV_TRBJ2-7*01
GGTCCAGGCACCAGGCTCACGGTC
```

Supplementary File S3.

>BV_TRBV01*01
ATAACTTTGCTGGAGCAGCAGCCTAGGTGGGGTCTGGTACGTCGTGGTCAAGCTGTGACC
CTGCGCTGTATCTTGAAGAATTCCCAGTACCCATGGCTGAGCTGGTACCAGCAGGATCTC
CAACAGCAACTACAATGGCTAGTCTCTCTGCGGAGTTCTGGGGACAAAGAGGCCAAATCT
CTCCCTGGTGCTGATTACCTGGCCACACGAGTCACGGATACAGAGCTGAGGCTCCAAGTG
ACCAACATGACCCAGAGCAGAACCTTGTATTGCACCTGC
>BV_TRBV01*02
ATAACTTTGCTGGAGCAGCAGCCTAGGTGGGGTCTGGTACGTCGTGGTCAAGCTGTGACC
CTGCGCTGTATCTTGAAGAATTCCCAGTACCCATGGCTGAGCTGGTACCAGCAGGATCTC
CAACAGCAACTACAATGGCTAGTCTCTCTGCGGAGTTTTGGGGACAAAGAGGCCAAATCT
CTCCCTGGTGCTGATTACCTGGCCACACGAGTCACTGATACAGAGCTGAGGCTCCAAGTG
ACCAACATGACCCAGAGCAGAACCTTGTATTGCACCTGC
>BV_TRBV01*03
ATAACTTTGCTGGAGCAGCAGCCTAGGTGGGGTCTGGTACGTCGTGGTCAAGCTGTGACC
CTGCGCTGTATCTTGAAGAATTCCCAGTACCCATGGCTGAGCTGGTACCAGCAGGATCTC
CAACAGCAACTACAATGGCTAGTCTCTCTGCGGAGTTCTGGGGACAAAGAGGCCAAATCT
CTCCCTGGTGCTGATTACCTGGCCACACGAGTCACTGATACAGAGCTGAGGCTCCAAGTG
ACCAACATGACCCAGAGCAGAACCTTGTATTGCACCTGC
>BV_TRBV01*04
ATAACGTTGCTGGAGCAACAGCCTAGGTGGGGTCTGGTACGTCGTGGTCAAGCTGTGACC
CTGCGCTGTATCTTGAAGAATTCCCAGTACCCATGGATGAGCTGGTACCAGCAGGATCTC
CAACAGCAACTACAATGGCTAGTCTCTCTGCGGAGTTCTGGGGACAAAGAGGCCAAATCT
CTCCCTGGTGCTGATTACCTGGCCACACGAGTCACTGATACAGAGCTGAGACTCCAAGTG
GCTAACATGACCCAGAGCAGAACCTTGTTTTGCACCTGC
>BV_TRBV01*05
ATAACGTTGCTGGAGCAGCAGCCTAGTTGGGGTCTGGTACGTCGTGGTCAAGCTGTGACC
CTGCGCTGTATCTTGAAGAATTCCCAGTACCCATGGATGAGCTGGTACCAGCAGGATCTC
CAACAGCAACTACAGTGGCTGTTCAGTCTGGGGAGTTCTGGGGACAAAGAGGCCAAATCT
CTCCCTGGTGCTGATTACCTGGCCACACGAGTCACTGATACAGAGCTGAGACTCCAAGTG
GCTAACATGACCCAGAGCAGAACCTTGTATTGCACCTGC
>BV_TRBV01*06
ATAACTTTGCTGGAGCAGCAGCCTAGTTGGGGTCTGGTACGTCGTGGTCAAGCTGTGACC
CTGCGCTGTATCTTGAAGAATTCCCAGTACCCATGGATGAGCTGGTACCAGCAGGATCTC
CAACAGCAACTACAGTGGCTGTTCAGTCTGGGGAGTTCTGGGGACAAAGAGGCCAAATCT
CTCCCTGGTGCTGATTACCTGGCCACACGAGTCACTGATACAGAGCTGAGACTCCAAGTG
GCTAACATGACCCAGAGCAGAACCTTGTATTGCACCTGC
>BV_TRBV02*01
GACCCTAGAATTACCCAGAAACCAAAACATGTGGTGGCAGTCACAGGGAATGAAAGGACC
CTGGAATGTCATCAGTATCTAGGTCACAATGCGATGTACTGGTATAGACAAAGCGCTAAG
AGGTCCCTAGAACTCATGTTTTCCTATAACTATCAAAATCTCGTTGAAAACCAGACTATC
CAAAGTCGCTTCCAGCCTCACAATCCAGAGAAATCCTCCTTAGACCTTCAGATCTCCGCC
CTGGAACCTGGCGACTCGGCCACATATTTCTGC
>BV_TRBV02*02
GACCCTAGAATTACCCAGAAACCAAAACATGTGGTGGCAGTCACAGGGAATGAAAGGACC
CTGGAATGTAATCAGTATCTAGGTCACAATGCGATGTACTGGTATAGACAAAGCGCTAAG
AGGTCCCTAGAGCTCATGTTTTCCTATAATTATCAAAATCTCGTTGAAAACCAGACTATC
CAAAGTCGCTTCCAGCCTCACAATCCAGAGAAATCCTTCTTAGACCTTCAGATCTCCGCC
CTGGAACCTGACGACTCGGCCACATATTTTTGC
>BV_TRBV02*03
GACCCTAGAATTACCCAGAAACCAAGACATGTGGTGGCAGTCACAGGGAATGAAAGGACC
CTGGAATGTCATCAGTATCTAGGTCACAATGCGATGTACTGGTATAGACAAAGCGCTAAG
AGGTCCCTAGAACTCATGTTTTCCTATAACTATCAAAATCTCGTTGAAAACCAGACTATC

CAAAGTCGCTTCCAGCCTCACAATCCAGAGAAATCCTCCTTAGACCTTCAGATCTCCGCC
CTGGAACCTGGCGACTCGGCCACATATTTCTGC
>BV_TRBV02*04
GACCCTAGAATTACCCAGAAACCAAAACATGTGGTGGCAGTCACAGGGAATGAAAGGACC
CTGGAATGTAATCAGTATCTAGGTCACAATGCGATGTACTGGTATAGACAAAGCGCTAAG
AGGTCCCTAGAGCTCATGTTTTCCTATAACTATCAAAATCTCGTTGAAAACCAGACTATC
CAAAGTCGCTTCAAGTCTCACAATCCAGAGAAATCCTACTTAGACCTTCAGATCTCCGCC
CTGGAACCTGACGACTCGGCCACGTATTTCTGC
>BV_TRBV03*01
GGACCCAACGTCTTGCAGTTCCCAAGTCATCAAGTGACACGTGAGGGGCAGATGGTGATC
CTCAGTTGTGACCCTGTCTCTAATCACCAGTCCTTCTATTGGTATAAACTGATCTTGGGA
CAAAACATAGAGTTTCTGGTTTCTTTCTACAGTGGTAAACCTATGGAAAAGTCTAAGCTA
TTCGAGGAGGATCGATTTTCAGTCGAAAGGCAAGAGGATTCATATTTCACTCTGAAGATA
CAGCCCACAACGCCGGAGGACTCAGCCGTGTACTTCTGT
>BV_TRBV03*02
GGACCCAACGTCTTGCAGTTCCCAAGTCATCAAGTGATACGTGAGGGGCAGATGGTGATC
CTCAGTTGTGACCCTGTCTCTAATCACCAGTCCTTCTATTGGTATAAACTGATCTTCGGA
CAAAACATAGAGTTTCTGGTTTCTTTCTACAATGGTAAACCTATGGAAAAGTCTAAGCTA
TTCGAGGAGGATCGATTTTCAGTCGAAAGGCAAGAGGGTTCATATTTCACTCTGAAGATA
CAGCCCACAACGCTGGAGGACTCAGCCGTGTACTTCTGT
>BV_TRBV03*03
GGACCCAGCGTCTTGCAGTTCCCAAGTCATCAAGTGACACGTGAGGGGCAGATGGTGATC
CTCAGTTGTGACCCTGTCTCTAGTCACCTGTACTTCTATTGGTATAAACTGATCTTGGGA
CAGAAGATAGAGTTTTTGGTTTCTTTTTACAATGGTAAACCTATGGAAAAGTCTAAGCTA
TTCGAGGAGGATCGATTTTCAGTCGAAAGGCAAGAGGATTCATATTTCACTCTGAAGATA
CAGCCCACAACGCCGGAGGACTCAGCCGTGTACTTCTGT
>BV_TRBV03*04
GGACCCAACGTCTTGCAGTTCCCAAGTCATCAAGTGACACGTGAGGGGCAGATGGTGATC
CTCAGTTGTGACCCTGTCTCTAGTCACCTGTACTTCTATTGGTATAAACTGATCTTGGGA
CAGAAGATAGAGTTTTTGGTTTCTTTCTACAATGGTAAACCTATGGAAAAGTCTAAGCTA
TTCGAGGAGGATCGATTTTCAGTCGAAAGGCAAGAGGATTCATATTTCACTCTGAAGATA
CAGCCCACAACGCCGGAGGACTCAGCCGTGTACTTCTGT
>BV_TRBV03*05
GGACCCAACGTCTTGCAGTTCCCAAGTCATCAAGTGATACATGAGGGGCAGATGGTGACC
CTCAGTTGTGACCCTGTCTCTAATCACCTGTACTTCTATTGGTATAAACTGATCTTGGGA
CAGAAGATAGAGTTTCTGGTTTCTTTCTACGATGGTAAACCTATGGATAAGTCTAAGCTA
TTCAAGGAGGATCGATTTTCAGTCGAAAGGCAAAAGGGTTCATATTTCACTCTGAAGATA
CAGCCCACAACGCTGGAGGACTCAGCCGTGTACTTCTGT
>BV_TRBV04*01
GAGACAACTGTTTTCCAGAATCCACAATATCTTGTCACGCAGGTGGGAAACAAAGCATCT
CTCAAGTGTGACCAAGATCTGGGCCATGATGCTATGTACTGGTACAAACAAGACTCTAAG
CAATCTCTGAAGATTATGTTTATCTACAATAGCAAGCAACTCATTTTAAATGAATCGGTT
CCAAGTCGCTTCTCACCTCAGTCTCCAGATAAAGCTCATCTGAATCTTCAAGTCAGGCTT
GTAGAACCGGATGACTCTGCTGTGTACTTCTGT
>BV_TRBV04*02
GAGACAACTGTTTTCCAGAATCCACAGTATCTTGTCACGCAGGTGGGAAATAAAGCATCT
CTCAAGTGTGACCAAGATCTGGGCCATGATGCTATGTACTGGTACAAACAAGACTCTAAG
CAATCTCTGAAGATTATGTTTATCTACAATAACAAGGAACTCATTTTAAATGAATCGGTT
CCAAGTCGCTTCTCACCTCAGTCTCCAGATAAAGCTCATCTGAATCTTCAAGTCAGGCTT
GTAGAACCGGATGACTCTGCTGTGTACTTCTGT
>BV_TRBV05*01
AACACTAAAATTGCCCAGACACCAAGATATCTGGTCATGGGAAGAGCAAATAATAAGTCT

TTGGATTGTAAACAACATCTGGGACATAATGCTATGTACTGGTATCAACAGAAAGCTGGG
AAGCCCCTAGAGCTCATGTTTCTCTACAATCTTAAAAACCTGATTCAAAATGAGACTGTG
CCCCTTCGTTTCTTACCTGAATGCCCAGACACCTCCCAGTTGTTCCTTCACCTGTCTGGC
CTGGAGCCAGAAGACTCAGCTGTGTACTGGTGT
>BV_TRBV05*02
AACACTAAAATTACCCAGACACCAAGATATCTGGTCATGGGAAGAGCAAATAATAAGTCT
TTGGATTGTAAACAACATCTGGGACATAATGCTATGTACTGGTATCAACAGAAAGCTGGG
AAGCCCCCAGAGCTCATGTTTCTCTACAATCTTAAAAACCTGATTCGAAATGAGACTGTG
CCCCTTCGTTTCTTACCTGAATGCCCAGACACCTCCCAGTTGTTCCTTCACCTGTCTGGC
CTGGAGCCAGAAGACTCAGCTGTGTACTGGTGT
>BV_TRBV05*03
AACACTAAAATTGCCCAGACACCAAGATATCTGGTCATGGGAAGAGCAAATAATAAGTCT
TTGGATTGTAAACAACATCTGGGACATAATGCTATGTACTGGTATCAACAGAAAGCTGGG
AAGCCCCTAGAGCTCATGTTTCTCTACAATCTTAAAAATCTGATTCAAAATGAGACTGTG
CCCCTTCGTTTCTTACCTGAATGCCCAGACACCTCCCAGTTGTTCCTTCACCTGTCTGGC
CTGGAGCCAGAAGACTCAGCTGTGTACTGGTGT
>BV_TRBV08*01
AGTGCTGGCATTATTCAGACTCCCAAATCGATGCTCGTGAAGACAGGACAGCATGTGTCA
ATGAAATGTACTCAGGATATGAAACATGACTCCATGTTCTGGTACCGACAGGATCCAGGG
CTGGGGCTGAGACTCATCTACGACTCATATGATGTTGGCATCACTGACAAAGGAGATGTC
CCTGAGGGGTACAATGTTTCCAGATCAAACACAAAGGACTTCTTCCTTATTCTGGAGTCA
GCCTCCCCCTCCCAGACATCTGTGTACTTCTGT
>BV_TRBV08*02
AGTGCTGGCATTATTCAGACTCCCAAATCGATGCTCGTGAAGACAGGACAGCATGTGTCA
ATGAAATGTACTCAGGATATGAAACATGACTCCATGTTCTGGTACCGACAGGATCCAGGG
CTGGGGCTGAGACTCATCTACTACTCATATGATGTTGGCATCACTGACAAAGGAGATGTC
CCTGAGGGGTACAATGTTTCCAGATCAAACAAAAAGGACTTCTTCCTTATTCTGGAGTCA
GCCTCCCCCTCCCAGACATCTGTGTACTTCTGT
>BV_TRBV09*01
AATGCTGGAGTTTCCCAATCTCCCTTGCACAGAATTATAAAGAGGGGACAAGATGTAGCC
CTTGAGTGTGATCCAGTTTCTGGCCACAGAGGTCTTTACTGGTACCGACAGAACCTGGGG
CAAGGACCAGTGTTCATGGTATCTTTCCAGGATGAGCTTTTATCAGACAAATCTGGGATG
CCTAATGATCGGTTCTCTGCAGGGAGGCCTGCGGGTTCCTCCTCCACTCTGAAGATCCAG
TCTACAGATCAAGAGGACTCGGGCATATACCTCTGT
>BV_TRBV10*01
GATGCTGGAATCACTCAGACACCTAGATACTGGATCGTGCTGACAGGAAGAAAGATGACC
CTGGAATGTTCTCAGAATATGAACCACTATTCGATGTTCTGGTATCGACAAAATGCGGGA
GAGGGATTGAAGCTCATCTATTACTCAGGAAGTGTTGGCAGCACTACCAAAGGAGATGTC
CCCGAAGGATACAGTGTCTCCAGAAACAAGAGCGAGCATTTCCCCCTGACATTAGGCTCA
GCCAGGCCCAGCCAGACCTCCACCTACTTCTGT
>BV_TRBV10*02
GATGCTGGAATCACTCAGACACCTAGATACTGGATCGTGCTGACAGGAAGAAAGATGACC
CTGGAATGTTCTCAGAATATGAACCACTATTCGATGTTCTGGTATCAACAAAATGCGGGA
GAGGAATTGAAGCTCATCTATTACTCAGGAAGTGTTGGCAGCACTACCAAAGGAGATGTC
CCAGAAGGATACAGTGTCTCCAGAAACAAGAGCGAGCATTTCCCCCTGGCATTAGGCTCA
GCCAGGCCCAGCCAGACCTCCACCTACTTCTGT
>BV_TRBV10*03
GATGCTGGAATCACTCAGACACCTAGATACTGGATCGTGCTGACAGGAAGAAAGATGACC
CTGGAATGTTCTCAGAATATGAACCACTATTCGATGTTCTGGTATCGACAAAATGCGGGA
GAGGGATTGAAGCTCATCTATTACTCAGGAAGTGTTGGCAGCACTACCAAAGGAGATGTC
CCAGAAGGATACAGTGTCTCCAGAAACAAGAGCGAGCATTTCCCCCTGGCATTAGGCTCA
GCCAGGCCCAGCCAGACCTCCACCTACTTCTGT

>BV_TRBV10*04
GATGCTGGAATCACTCAGACACCTAGATACTGGATCGTGCTGACAGGAAGAAAGATGACC
CTGGAATGTTCTCAGAATATGAACCACTATTGGATGTTCTGGTATCGACAAGATGCGGGA
GAGGGACTGAAGCTGATCTATTACTCAGGAAGTGTTGGCAGCACTACCAAAGGGGATGTC
CCAGAAGGATACAGTGTCTCCAGAAACAAGAGCGAGCATTTCCCACTGGCATTAGGCTCA
GCCAGGCCCAGCCAGACCTCCACCTACTTCTGT
>BV_TRBV10*05
GATGCTGGAATCACTCAGACACCTAGATACTGGATCGTGCTGACAGGAAGAAAGATGACC
CTGGAATGTTCTCAGAATATGAACCACTATTGGATGTTCTGGTATCGACAAGATGCGGGA
GAGGGACTGAAGCTGATCTATTACTCAGGAAGTGTTGGCAGCACTACCAAAGGGGATGTC
CCAGAAGGATACAGTGTCTCCAGAAACAAGAGCGAGCATTTCCCGCTGGCATTAGGCTCA
GCCAGGCCCAGCCAGACCTCCACCTACTTCTGT
>BV_TRBV1201*01
AGTCCCGGGGTCATCCAGTCTCCAAGACACATAATCAAAGCAAAGGGAGGAAGGTCCACT
CTCAAATGTACTCCCATCTCTGGACATGATACTGTGTTCTGGTATCAGCAATCTCAGGGG
AAGGAACTGAAGTTCCTTATTCAACACTTTGAAAAAATGGAGAGAGCGAAAGGAAACATA
CCCAACCGATTCTCAGTCCAGCAGTTCAGTGACTATCACTCTGAGATGAACATGAGCGCC
TTGCAGCTGGAGGACTCTGCTGTGTACTTCTGT
>BV_TRBV1201*02
AGTCCTGGGGTCATCCAGTCTCCAAGACACATAATCAAAACAAAGGGAGGAAGGTCCACT
CTCAAATGTACTCCCATCTCTGGACATGACAGTGTGGGCTGGTACCAGCAATCTCAGGGG
AAGGAACTGAAGTTCCTTATTCAACACTATGAAAAAATGGAGAGAGCGAAAGGAAACATA
CCCAACCGATTCTCAGTCCAGCAGTTCAGTGACTATCACTCTGAGATGAACATGAGCGCC
TTGCAACTGGAGGACTCTGCTGTGTACTTCTGT
>BV_TRBV1201*03
AGTCCCGGGGTCATCCAGTCTCCAAGACACATAATCAAAACAAAGGGAGGAAGGTCCACT
CTCAAATGTACTCCCATCTCTGGACATGACAGTGTGGGCTGGTACCAGCAATCTCAGGGG
AAGGAACTGAAGTTCCTTATTCAACACTATGAAAAAATGGAGAGAGAGAAAGGAAACATA
CCCAGCCGATTCTCAGTCCAGCAGTTTAGTGACTATCACTCTGAGATGAACATGAGCGCC
TTGCAGCTGGAGGACTCTGCTGTGTACTTCTGT
>BV_TRBV1201*04
AGTCCCGGGGTCATCCAGTCTCCAAGACACATAATCAAAGCAAAGGGAGGAAGGTCCACT
CTCAAATGTACTCCCATCTCTGGACATGATACTGTGTTCTGGTATCAGCAATCTCAGGGG
AAGGAACTGAAGTTCCTTATTCAACACTTTGAAAAAATGGAGAGAGCGAAAGGAAACATA
CCCAACCGATTCTCAGTCCAGCAGTTCAGTGACTCTCACTCTGAGATGAACATGAGCGCC
TTGCAGCTGGAGGACTCTGCTGTGTACTTCTGT
>BV_TRBV1202*01
AGTCCCGGGGTCGTCCAGTCTCCAAGATACATAATCAAAACAAAGGGAGGAAAGTCCTTT
CTCAAATGTACTCCCATCTCTGGACAGGACAATGTGTTCTGGTACCAGCAGACTCTGGGG
CAGAGACTGAAGTTCCTCATTCAACACTATAAAAGAATGGAGAGAGCGAAAGGAAACATA
CCCAGCCGATTCTCAGTCCAGCAGTTCAGTGACTCTCACTCTGAGATGAACATGAGCGCC
TTGCAGCTGGAGGACTCTGGTGTGTACTTCTGT
>BV_TRBV1202*02
AGTCCCGGGGTCGTCCAGTCTCCAAGATACATAATCAAAACAAAGGGAGGAAGGTCCTTT
CTCAAATGTACTCCCATCTCTGGACAGGACAATGTGTTCTGGTACCAGCAGACTCTGGGG
CAGAAACTGAAGTTCCTCATTCAACACTATAAAAGAATGGAGAGAGCGAAAGGAAACATA
CCCAACCGATTCTCAGTCCAGCAGTTCAGTGACTCTCACTCTGAGATGAACATGAGCGCC
TTGCAGCTGGAGGACTCTGCTGTGTACTTCTGT
>BV_TRBV1202*03
AGTCCCGGGGTCGTCCAGTCTCCAAGATACATAATCAAAACAAAGGGAGGAAGGTCCTTT
CTCAAATGTACTCCCATCTCTGGACAGGACAATGTGTTCTGGTACCAGCAGACTCTGGGG
CAGAGACTGAAGTTCCTCATTCATCACTATAAAAGAATGGAGAGAGCGACAGGAAACATA

TCCAAACGATTCTCAGTTCAGCAGTTCAGTGACTCTCACTCTGAGATGAACATGAGCGCC
TTGCAGCTGGAGGACTCTGCTGTGTACTTCTGT
>BV_TRBV1203*01
AGTCCCGGGGTCGTCCAGTCTCCAAGACACATAATCAAAGCAAAGGGAGGAAGGTCCTTT
GTGAAATGTACTCCCATCGATGGACATAACACTGTGTTCTGGTACCAGCAGACTCTGGGG
CAGAAACTGAAGTTCCTCATTCAACACTATGAAAAAATGGAGAGAGAGAAAGGAAACATA
CCCAGTCGATTCTCAGTCCAGCAGTTCAGTGACTATCACTCTGAGATGAACATGAGCGCC
TTGCAGCTGGAGGACTCTGCTGTGTACTTCTGT
>BV_TRBV1203*02
AGTCCCGGGGTCGTCCAGTCTCCAAGACACATAATCAAAGCAAAGGGAGGAAGGTCCTTT
GTGAAATGTACTCCCATCGATGGACATAACACTGTGTTCTGGTACCAGCAGACTCTGGGG
CAGGAACTGAAGTTCCTCATTCAACACTATGAAAAAATGGAGAGAGAGAAAGGAAACATA
CCCAGTCGATTCTCAGTCCAGCAGTTCAGTGACTATCACTCTGAGATGAACATGAGCGCC
TTGCAGCTGGAGGACTCTGCTGTGTACTTCTGT
>BV_TRBV1203*03
AGTCCCAGGGTCATCCAGTCTCCAAGACACATAATCAAAGCAAAGGGAGGAAGGTCCTTT
GTGAAATGTACTCCCATCGATGGACATAACACTGTGTTCTGGTACCAGCAGACTCTGGGG
CAGGAACTGAAGTTCCTCATTCAACACTATGAAAAAATGGAGAGAGCGAAAGGAAACATA
CCCAGTCGATTCTCAGTTCAGCAGTTCAGTGACTATCACTCTGAGATGAACATGAGCGCC
TTGCAGCTGGAGGACTCTGCTGTGTACTTCTGT
>BV_TRBV1203*04
AGTCCCGGGGTCGTCCAGTCTCCAAGACACATAATCAAAGCAAAGGGAGGAAGGTCCTTT
GTGAAATGTACTCCCATCGATGGACATAACACTGTGTTCTGGTACCAGCAGACTCTGGGG
CAGGAACTGAAGTTCCTCATTCAACACTATGAAAAAATGGAGAGAGAGAAAGGAAACATA
CCCAGCCGATTCTCAGTCCAGCAGTTCAGTGACTATCACTCTGAGATGAACATGAGCGCC
TTGCAGCTGGAGGACTCTGCTGTGTACTTCTGT
>BV_TRBV1204*01
AGTCCCGGGGTCGTCCAGTCTCCAAGACACATAATCAAAGCAAAGGGAGGAAGGTCCTTT
GTGAATTGTACTCCCATCTCTGGACATGCCAGTGTGTTCTGGTACCAGCAGACTCTGGGC
CAGGAACTGAAGTTCCTCATTCAGCATTATGAAAAAATGGAAAGAGAGAAAGGAAATATA
CCCAACCGATTCTCAGTCCAGCAGTTCAGTGACTATCACTCTGAGATGAACATGAGCACC
TTGCAGCTGGAGGACTCGGCGGTGTACCTCTGC
>BV_TRBV1204*02
AGTCCCGGGGTCGTCCAGTCTCCAAGACACATAATCAAAGCAAAGGGAGGAAGGTCCTTT
GTGAATTGTACTCCCATCTCTGGACATGCCAGTGTGTACTGGTACCAGCAGACTCTGGGC
CAGGAACTGAAGTTCCTCATTCAGCATTATGAAAAAATGGAAAGAGAGAAAGGAAATATA
CCCAACCGATTCTCAGTCCAGCAGTTCAGTGACTATCACTCTGAGATGAACATGAGCACC
TTGCAGCTGGAGGACTCGGCGGTGTACCTCTGC
>BV_TRBV1204*03
AATCCCGGGGTCGTCCAGTCTCCAAGACACATAATCAAAGCAAAGGGAGGAAGGTCCTTT
GTGAATTGTACTCCCATCTCTGGACATGCCAGTGTGTACTGGTACCAGCAGACTCTGGGC
CAAGAACTGAAGTTCCTCATTCAGCATTATGAAAAAATGGAAAGAGAGAAAGGAAATATA
CCCAACCGATTCTCAGTCCAGCAGTTCAGTGACTATCACTCTGAGATGAACATGAGTGCC
TTGGAGCTGGAGGACTCGGCGGTGTACCTCTGC
>BV_TRBV1205*01
AGTCCCGGGGTCATCCAGTCTCCAAGACACATAATCAAAGCAAAGGGAGGAAGGTCCTTT
ATGAATTGTACTCCCATCTCTGGACATGATACTGTGTTCTGGTACCAGCAGACTCTGGGG
CAGGAACTGAAGTTCCTCATTCAGCATTATCAAAAAACGGAGGGAGAGAAAGGAAACATA
CCCAGCCGATTCTCAGTCCAGCAGTTCAGTGACTATCACTCTGAGATGAACATGAGCGCC
TTGCAGCTGGAGGACTCTGCTGTGTACTTCTGT
>BV_TRBV1205*02
AGTCCCGGGGTCATCCAGTCTCCAAGACACATAATCAAAGCAAAGGGAGGAAGGTCCTTT

ATGAATTGTACTCCCATCTCTGGACATGATACTGTGTTCTGGTACCAGCAGACTCTGGGG
CAGGAACTGAAGTTCCTCATTCAGCATTATCAAAAAACGGAGGGAGAGAAAGGAAACATA
CCCAGCCGATTCTCAGTCCAGCAGTTCAGTGACTATCACTCTGAGATGAACATGAGCGCC
TTGCAGCTGGAGGACTCTGGTGTGTACTTCTGT
>BV_TRBV1206*01
AGTCCCGGGGTCGTCCAGTCTCCAAGACACATAATCAAAGCAAAGGGAGGAAGGTCCTTT
GTGAATTGTACTCCCATCTCTGGACATAACACTGTGTTCTGGTACCAGCAGACTCTGGGG
CAGGAACTGAAGTTCCTCATTCGGCATTATCAAGAAATGGAAGGAGAGAAAGGAAACATA
CCCAGCCGATTCTCAGTCCAGCAGTTTAGTGACTATCACTCTGAGATGAACATGAGCGCC
TTGCAGCTGGAGGACTCTGCCGTGTACTTCTGT
>BV_TRBV1206*02
AGTTCCGGGGTCATCCAGTCTCCAAGACACATAATCAAAGCAAAGGGAGGAAGGTCCTTT
GTGAATTGTACTCCCATCTCTGGACATAACACTGTGTTCTGGTACCAGCAGACTCTGGGG
CAGGAACTGAAGTTCCTCATTCGGCATTATCAAGAAATGGAGGGAGAGAAAGGAAACATA
CCCAGCCGATTCTCAGTCCAGCAGTTTAGTGACTATCACTCTGAGATGAACATGAGCGCC
TTGCAGCTGGAGGACTCTGCCGTGTACTTCTGT
>BV_TRBV1206*03
AGTCCCGGAGTCGTCCAGTCTCCAAGACACATAATCAAAGCAAAGGGAGGAAGGTCCTTT
GTGAATTGTACTCCCATCTCTGGACATGATACTGTGTTCTGGTACCAGCAGACTCTGGGC
CAGGAACTGAAGTTCCTCATTCGGCATTATCAAGAAATGGAGGGAGAGAAAGGAAACATA
CCCAACCGATTCTCAGTCCAGCAGTTCAGTGACTATCACTCTGAGATGAACATGAGCGCC
TTGCAGCTGGAGGACTCTGCCGTGTACTTCTGT
>BV_TRBV1206*03
AGTCCCGGGGTCATCCAGTCTCCAAGACACATAATCAAAGCAAAGGGAGGAAGGTCCTTT
GTGAATTGTACTCCCATCTCTGGACATAACACTGTGTTCTGGTACCAGCAGACTCTGGGG
CAGGAACTGAAGTTCCTCATTCGGCATTATCAAGAAATGGAGGGAGAGAAAGGAAACATA
CCCAGCCGATTCTCAGTCCAGCAGTTCAGTGACTCTCACTCTGAGATGAACATGAGCGCC
TTGCAGCTGGAGGACTCTGCTGTGTACTTCTGT
>BV_TRBV1207*01
AGTCCCGGGGTTGTCCAGTCTCCAAGACACATAATCAAAGCAAAGGGAGGAAAGTCCTTT
GTGAATTGTACTCCCATCTCTGGACATAACACTGTGTTCTGGTATCAGCAGACTCTGGGG
CAGGAACTGAAGTTCCTCATTCAGCATTTTGAAAAAATAGAGAGCGAGAAAGGAAACATA
CCCAACCGATTCTCAGTCCAGCAGTTCAGTGACTATCACTCTGAGATGAACATGAGCGCC
TTGCAGCTGGAGGACTCTGCTGTGTACTTCTGT
>BV_TRBV1301*01
GAGGCTGCAATCACCCAAAGCCCGAGAAGCAAGATGACAGTAACAGGAGGAAAGGTGGAA
CTAAGCTGTAACCAGACAGACAACCACAATTATATGTACTGGTATCGGCAGGACCTGGGT
CATGGACTGAGGCTGATCCATTACTCATTTGGTGCTGGCAGCACTGAGAACGGAGATGTC
CCTGATGGGTACAAGGCCATTAGACCAAGCCAAGAGGAGTTCTCTCTCATTCTGGAGAAG
GCTTCCCCCTCTCAGACAGCTGTGTACTTCTGT
>BV_TRBV1301*02
GAGGCTGCAATCACCCAAAGCCCGAGAAGCAAGATGACAGTAACAGGAGGAAAGGTGGAA
CTAAGCTGTAACCAGACAGACAACCACAATTATATGTACTGGTATCGGCAGGACCTGGGT
CATGGACTGAGGCTGATCCATTACTCATTTGGTGCTGGCAGCACTGAGAACGGAGATGTC
CCTGATGGGTACAAGGCCATTAGACCAAGCCTAAAGGATTTCTCTCTCATTCTGGAGAAG
GCTTCCCCCTCTCAGACAGCTGTGTACTTCTGT
>BV_TRBV1301*03
GAGGCTGCAATCACCCAAAGCCCGAGAAGCAAGATGACAGTAACAGGAGGAAAGGTGGAA
CTAAGCTGTAACCAGACAGACAACCACAACATTATGTACTGGTATCGGCAGGACCTGGGT
CATGGACTGAGGCTGATCCATTACTCATTTGGTGCTGGCAGCACTGAGAACGGAGATGTC
CCTGATGGGTACAAGGCCATTAGACCAAGCCTAAAGGATTTCTCTCTCATTCTGGAGAAG
GCTTCCCCCTCTCAGACAGCTGTGTACTTCTGT

>BV_TRBV1302*01
GAGGCTGCAGTCACCCAAAGCCCGAGAAACAAGGTGACAGTAACAGGAGGAAAGGTGGAA
CTAAGCTGTCACCAGACGGACAACCACAACATTATGTACTGGTATCGGCAGGACCTGGGT
CATGGACTGAGGCTGATCCATTACTCATATGGTGATGGCAGCACTGAGAACGGAGATGTC
CCTGATGGGTACAAGGCCACCAGACCGAAGACAGAGGAGTTCTCTCTCATTCTGGAGAAG
GCTTCCCCCTCTCAGACAGCTGTGTACTTCTGT
>BV_TRBV1302*02
GAGGCTGCAGTCACCCAAAGCCCGAGAAACAAGGTGACAGTAACAGGAGGAAAGGTGGAA
CTAAGCTGTCACCAGACGGACAACCACAACATTATGTACTGGTATCGGCAGGACCTGGGT
CATGGACTGAGGCTGGTCCATTACTCATATGGTGATGGCAGCACTGAGAACGGAGATGTC
CCTGATGGGTACAAGGCCACCAGACCGAAGACAGAGGAGTTCTCTCTCATTCTGGAGAAG
GCTTCCCCCTCTCAGACAGCTGTGTACTTCTGT
>BV_TRBV1302*03
GAGACTGCAGTCACCCAAAGCCCGAGAAACAAGGTGACAGTAACAGGAGGAAAGGTGGAA
CTAAGCTGTCACCAGACGGACAACCACAACAATATGTACTGGTATCGGCAGGACCTGGGT
CATGGACTGAGGCTGATCCATTACTCATATGGTGATGGCAGCACTGAGAACGGAGATGTC
CCTGATGGGTACAAGGCCACCAGACCGAAGACAGAGGAGTTCTCTCTCATTCTGGAGAAG
GCTTCCCCCTTTCAGACATCTGTGTACTTCTGT
>BV_TRBV1303*01
GAGGCTGCAGTCACCCAAAGCCCAAGAAGCAAGGTGACAGTAACAGGAGGAAAGGTGGAA
CTAAGCTGTAACCAGACGGACAACCACAACAATATGTACTGGTATCGTCAGGACCTGGGT
CATGGACTGAGGCTGATCTATTACTCATATGGTGCTGACAACATTCAAAAAGGAGATGTC
CCTGATGGGTACAAGGTCATCAGACCAAGGACAGAGGAGTTCTCTCTTATTCTGGAGAAG
GCTTCCCCCTCTCAGACAGCTGTGTACTTCTGT
>BV_TRBV1303*02
GAGGCTGCAGTCACCCAAAGCCCAAGAAGCAAGGTGACAGTAACAGGAGGAAAGGTGGAA
CTAAGCTGTCACCAGACGGACAACCACAACAATATGTACTGGTATCGTCAGGACTTGGGT
CATGGACTGAGGCTGATCTATTACTCATATGGTGCTGACAACATTCAAAAAGGAGATGTC
CCTGATGGGTACAAGGTCATCAGACCAAGGACAGAGGAGTTCTCTCTTATTCTGGAGAAG
GCTTCCCCCTCTCAGACAGCTGTGTACTTCTGT
>BV_TRBV1304*01
GAGGCTGCAGTCACCCAAAGCCCAAGAAGCAAGGTGACAGTAACAGGAGGAAAGGTGGAA
CTAAGCTGTCACCAGACTTTCAACCATGAAAATATGTACTGGTATCGGCAGGACCTGGGC
CATGGACTGAGGCTGATCCATTACTCATATGTTGCTGGTAGCACTGAGAACGGAGATGTC
CCTGATGGGTACAAGGCCACCAGACCAAGGACAGAGGAGTTCTCTCTCATTCTGGAGAAG
GCTTCCCCCTCTCAGACATCTGTGTACTTCTGT
>BV_TRBV1304*02
GAGGCTGCAGTCACCCAAAGTCCAAGAAGCAAGGTGACAGTAACAGGAGGAAAGGTGGAA
CTAAGCTGTAACCAGACTTTCAACCATGAAAATATGTACTGGTATCGGCAGGACCTGGGT
CATGGACTGAGGCTGATCCATTACTCATATGGTGCTGGTAGCACTGAGAACGGAGATGTC
CCTGATGGGTACAAGGCCACCAGACCAAGGACAGAGGAGTTCTCTCTCATTCTGGAGAAG
GCTTCCTCCTCTCAGACATCTGTGTACTTCTGT
>BV_TRBV1304*03
GAGGCTGCAGTCACCCAAAGCCCAAGAAGCAAGGTGACAGTAACAGGAGGAAAGGTGGAA
CTAAGCTGTAACCAGACTTTCAACCATGAAAATATGTACTGGTATCGGCAGGACCTGGGT
CATGGACTGAGGCTGATCCATTACTCATATGGTGCTGGTAGCACTGAGAACGGAGATGTC
CCTGATGGGTACAAGGCCACCAGACCAAGGACAGAGGAGTTCTCTCTCATTCTGGAGAAG
GCTTCCTCCTCTCAGACATCTGTGTACTTCTGT
>BV_TRBV1304*04
GAGGCTGCAGTCACCCAAAGCCCAAGAAGCAAGGTGACAGTAACAGGAGGAAAGGTGGAA
CTAAGCTGTAACCAGACTTTCAACCATGAAAATATGTACTGGTATCGGCAGGACCTGGGT
CATGGACTGAGGCTGATCCATTACTCATATGGTACTGGTAGCACTGAGAACGGAGATGTC

CCTGATGGGTACAAGGCCACCAGACCAAGGACAGAGGAGTTCTCTCTCATTCTGGAGAAG
GCTTCCTCCTCTCAGACATCTGTGTACTTCTGT
>BV_TRBV1305*01
GAGGCTGCAGTCACCCAAAGCCCAAAAAGCAAGGTGACAGTAACAGGAGGAAAGGTGGAA
CTAAGCTGTAACCAGACTTTCAAACATGACAATATGTACTGGTATCGGCAGGACCTGGGT
CATGCACTGAGGCTGATCTATTACTCATATGGTGTTGACAGCATCGAGAAAGGAGATGTC
CCTGATGGGTACAAGGTCACCAGACCAAGGACAGAGGAGTTCTCTCTCATTCTGGAGAAG
GCTTCCCCCTCTCAGACAGCTGTGTACTTCTGT
>BV_TRBV1305*02
TACGCGGGAGTCACCCAAAGCCCAAAAAGCAAGGTGACAGTAACAGGAGGAAAGGTGGAA
CTAAGCTGTAACCAGACTTTCAAACATGACAATATGTACTGGTATCGGCAGGACCTGGGT
CATGCACTGAGGCTGATCTATTACTCATATGGTGTTGACAGCATCGAGAAAGGAGATGTC
CCTGATGGGTACAAGGTCACCAGACCAAGGACAGAGGAGTTCTCTCTCATTCTGGAGAAG
GCTTCCCCCTCTCAGACAGCTGTGTACTTCTGT
>BV_TRBV1305*03
GAGGCTGCAGTCACCCAAAGCCCAAAAAGCAAGGTGACAGTAACAGGAGGAAAGGTGGAA
CTAAGCTGTAACCAGACTTTCAAACATGACAATATGTACTGGTATTGGCAGGACCTGGGT
CATGCACTGAGGCTGATCTATTACTCATATGGTGTTGACAGCATCGAGAAAGGAGATGTC
CCTGATGGGTACAAGGTCACCAGACCAAGGACAGAGGAGTTCTCTCTCATTCTGGAGAAG
GCTTCCCCCTCTCAGACAGCTGTGTACTTCTGT
>BV_TRBV1306*01
GAGGCTGCAGTCACTCAAAGCCCAAGAAGCAAGGTGACAATAACAGGAGGAAAGGTGGAA
CTAAGCTGTCACCAGACTGATGGCCATGACTATATGTACTGGTATCGGCAGGACCTGGGT
CATGCACTGAGGCTGATCTATTACTCATATGGTGTTGACAGCATCGAGAAAGGAGATGTC
CCTGATGGGTACAAAGTCACCAGACCAAGGACAGAGGACTTCTCTCTCATTCTGGAGAAG
GCTTCCCCCTCTCAGACAGCTGTGTACTTCTGT
>BV_TRBV1307*01
GAGGCTGCAGTCACCCAAAGCCCAAGAAGCAAGGTGACAGTAACAGGAGGAAAGGTGGAA
CTAAGCTGTAACCAGACTTTCAACCATGGCAATATGTACTGGTATCGTCAGGACCTGGGT
CATGGACTGAGGCTGATCTATTACTCATATGGTGCTGACAACATTCAAAAAGGAGATGTC
CCTGATGGGTACAAGGTCATCAGACCAAGGACAGAGGACTTCTCTCTTATTCTGGAGAAG
GCTTCCCCCTCTCAGACAGCTGTGTACTTCTGT
>BV_TRBV14*01
GAAGCTGGAGTCACTCAGTCTCCCAGATATGTGATCATACAGGAAAGACAAGAAGTTTCC
TTTTGGTGTGACCCTATTTCTGGACATAATACCCTTTACTGGTATCAGCAGCCCAGAGAC
CAGGGCCTCCAGTTTCTAACTTACTTTCAGAATGAGGCTATTATGGATGATTCACAACTG
CCCAAGGATCGTTTTTCTGCAGTGCGGCCCAACGGAGTCAACTCTACTCTGAAGATCCAG
TCTGCAAAGCTGGGGGACTCAGCCACATATCTCTGT
>BV_TRBV14*02
GAAGCTGGAGTCACTCAGTCTCCCAGATATGTGATCATACGGGAAAGACAAGAAGTTTCC
TTTTGGTGTGACCCTATTTCTGGACATAATACCCTTTACTGGTATCAGCAGCCCAGAGAC
CAGGGCCTCCAGTTTCTAACTTACTTTCAGAATGAGGCTATTATGGATGATTCACAACTG
CCCAAGGATCGTTTTTCTGCAGTGCGGCCTAACGGAGTCAACTCTACTCTGAAGATCCAG
TCTGCAAAGCTGGGGGACTCAGCCACATATCTCTGT
>BV_TRBV14*03
GAAGCTGGAGTCACTCAGTCTCCCAGATATGTGATCATACAGGAAAGACAAGAAGTTTCC
TTTTGGTGTGACCCTATTTCTGGACATACTACCCTTTACTGGTATCAGCAGCCCAGAGAC
CAGGGCCTCCAGTTTCTAACTTACTTTCAGAATGAGGCTATTATGGATGATTCACAACTG
CCCAAGGATCGTTTTTCTGCAGTGCGGCCCAACGGAGTCAACTCTACTCTGAAGATCCAG
TCTGCAAAGCTGGGGGACTCAGCCACATATCTCTGT
>BV_TRBV14*04
GAAGCTGGAGTCACTCAGTCTCCCAGATATGTGATCATACAGGAAAGACAAGAAGTTTCC

TTTTGGTGTGACCCTATTTCTGGACATAATACCCTTTACTGGTATCAGCAGTCCAGGGAC
CAGGGCCTCCAGTTTCTAACTTACTTTCAGAATGAGGCTATTATGGATGATTCACAACTG
CCCAAGGATCGTTTTTCTGCAGTGCGGCCTAACGGAGTCAACTCTACTCTGAAGATCCAG
TCTGCAAAGCTGGGGGACTCAGCCACATATCTCTGT
>BV_TRBV15*01
GATGCTGGAGTTACCCAGTCACCCAGATATGAGGTGGCAGAGAAAGGACAAACAGTAACT
CTGACTTGTGAACCAGTTTCAGGTCACAATGATCTTTTCTGGTACAGACAGACCAAGACA
CAGGGACTAGAGTTGTTGAGGTACTTTCGCAGCAAGTCCCTTATGGAGGACGAAAAGACA
GGAAAGGACAGATTCACAGCTGAGATGCTAAATTCATCTGCCTCCACTCTGAAGATCCAG
CCCACAGAACCCCAGGACTCAGCTGTGTATCTATGT
>BV_TRBV15*02
GATGCTGGAGTCACCCAGTCACCCAGACATGAGGTGGCAGAGAAAGGACAAACAGTAACT
CTGACTTGTGAACCAGTTTCAGGTCACAATGATCTTTTCTGGTACAGACAGACCAAGACA
CAGGGACTAGAGTTGCTGAGGTACTTTCGCAGCAAGTCCCTTATGGAGGACGAAAAGACA
GGAAAGGACAGATTCACAGCTGAGATGCTAAATTCATCTGCCTCCACTCTGAAGATCCAG
CCCACAGAACCCCAGGACTCAGCTGTGTATCTATGT
>BV_TRBV15*03
GATGCTGGAGTCACCCAGTCACCCAGACATGAGGTGGCAGAGAAAGGTCAAACAGTAACT
CTGACTTGTGAACCAGTTTCAGGTCACAATGATCTTTTCTGGTACAGACAGACCAAGACA
CAGGGACTAGAGTTGCTGAGGTACTTTCGCAGCAAGTCCCTTATGGAGGACGACAAGACA
GGAAAGAACAGATTCACAGCTGAGATGCTAAATTCATCTGCCTCCACTCTGAAGATCCAG
CCCACAGAACCCCAGGACTCAGCTGTGTATCTATGT
>BV_TRBV16*01
AATGCTGGGGTCACCCAAACACCCAGGCATAAGGTGACAGAGATGGGACAAGAAGTAACT
CTGAGATGTGAACCAATTTCAGGACACAGTGGTGTTTTCTGGTACAGGCAGACGACTGTG
CGGGGACTGGAATTTCTGATTTCCTTTCTCAACCAGGCTCCTATAGGTGAAACAGGGATG
CCCAAAGAACGTTTCTCAGCTCAGATGCCTAATGCGACGCTCTCCACTCTGAAGGTTGAG
ACCACACAACCCCAGGACTCAGCTGTGTATCTCTGT
>BV_TRBV16*02
AATGCTGGGGTCACCCAAACACCCAGGCATAAGGTGACAGAGATGGGACAAGAAGTAACT
CTGAGATGTGAACCAATTTCAGGACACAGTGGTGTTTTCTGGTACAGGCAGACGACTGTG
CGGGGACTGGAATTTCTGATTTCCTTTCTCAACCAGGCTCCTATAGGTGAAACAGGGATG
CCCAAAGAACGTTTCTCAGCTCAGATGCCTAATGCGACACTCTCCACTCTGAAGGTTGAG
ACCACACAACCCCAGGACTCAGCTGTGTATCTCTGT
>BV_TRBV16*03
AATGCTGGGGTCACCCAAACACCCAGGCATAAGGTGACAGAGATGGGACAAGAAGTAACT
CTGAGATGTGAACCAATTTCAGGACACAGTGGTGTTTTCTGGTACAGGCAGACGACTGTG
CGGGGACTGGAATTCCTGATTTCCTTTAGTAACCAGGCTCCGATAGATGAAACAGGGATG
CCCAAAGAACGTTTCTCAGCTCAGATGCCTAATGCGACGCTCTCCACTCTGAAGGTTGAG
ACCACACAACCCCAGGACTCAGCTGTGTATCTCTGT
>BV_TRBV16*04
AATGCTGGGGTCACCCAAACACCCAGGCATAAGGTGACAAAGATGGGACAAGAAGTAACT
CTGAGATGTGAACCAATTTCAGGACACAGTGGTGTTTTCTGGTACAGGCAGACGACTGTG
CGGGGACTGGAATTTCTGATTTCCTTTCTCAACCAGGCTCCTATAGGTGAAACAGGGATG
CCCAAAGAACGTTTCTCAGCTCAGATGCCTAATGCGACGCTCTCCACTCTGAAGGTTGAG
ACCACACAACCCCAGGACTCAACTGTGTATCTCTGT
>BV_TRBV16*05
AATGCTGGGGTCACCCAAACACCCAGGCATAAGGTGACAAAGATGGGACAAGAAGTAACT
CTGAGATGTGAACCAATTTCAGGACACAGTGGTGTTTTCTGGTACAGGCAGACGACTGTG
CGGGGACTGGAATTCCTGATTTCCTTTCTCAACCAGGCTCCTATAGGTGAAACAGGGATG
CCCAAAGAACGTTTCTCAGCTCAGATGCCTAATGTGACGCTCTCCACTCTGAAGGTTGAG
ACCACACAACCCCAGGACTCAGCTGTGTATCTCTGT

>BV_TRBV17*01
GATGCGATGGTCATGCAAAACCCAAGATACAAGGTTTCCAGGGTTGGAAAACCAGTGAAT
CTGAGCTGCTCCCAGAATCTGAATCATAATGCCATGTACTGGTACCAACAGAAACCAAAC
CAAGCACCAAAGCTTCTGCTCTACTACTATGATACAACATTGAACAGGGAGTCTGACACT
TCTGAAAACTTCCAATCCAACCGGCCCAACACTTCTTTCTGCTCTCTTGGCATTCACTCT
GCAGGCCTAGGGGATTCTTCCATGTACCTCTGT
>BV_TRBV17*02
GATGCGATGGTCATGCAAAACCCAAGATACAAGGTTTCCAGGGTTGGAAAACCAGTGAAT
CTGAGCTGCTCCCAGAATTTGAATCATGATGTCATGTACTGGTACCAACAGAAACCAAAC
CAAGCACCAAAGCTTCTGCTCTACTACTATGATACAACATTGAACAGAGAGGCTGACACT
TCTGAAAACTTCCAATCCAACCGGCCCAACACTTCTTTCTGCTCTCTTGGCATTCACTCT
GCAGGCCTAGGAGATTCTTCCTTGTACCTCTGT
>BV_TRBV17*03
GATGCAATGGTCATGCAGAACCCAAGATACAAGGTTGCCAGGGTTGGAAAACCAGTGAAT
CTGAGCTGCTCCCAGAATCTGAATCATAATGCCATGTACTGGTACCAACAGAAACCAAAC
CAAGCACCAAAGCTTCTGCTCTACTACTATGATACAACATTGAACAGAGAGGCTGACACT
TCTGAAAACTTCCAATCCAACCGGCCCAACACTTCTTTCTGCTCTCTTGGCATTCACTCT
GCAGGCCTAGGGGATTCTTCCATGTACCTCTGT
>BV_TRBV19*01
CACGGTGCCATCATTCAGAAACCCAAATTCCTGATCGGACTGCAAGGGCAGAAACTGACG
ATGGAATGCCAACAGACTTTTGATCACGATACCATGTACTGGTACCGGCAGGATCCAGGG
AAAGGGTTGAGACAGATCTACTATTCAATAACTGAAAAAGATATCCAGAAAGGGGAACTA
TCTGAAGGCTATAGCGTCTCTCGTGAGAAAAAGCCGTTTTTCCCTCTTACTGTGACATCT
GCCCAGAAGAACCAGATGGCTGTCTACCTCTGT
>BV_TRBV19*02
CACGGTGCCATCATTCAGAAACCCAAATTCCTGATCGGACTGCAAGGGCAGAAACTGACG
ATGGAATGCCAACAGACTTTTAATCACGATACCATGTACTGGTACCGGCAGGATCCAGGG
AAAGGGTTGAGACAGATCTACTATTCAATAACTGAAAAAGATATCCAGAAAGGGGAACTA
TCTGAAGGCTATAGCGTCTCTCGTGAGAAAAAGCCGTTTTTCCCTCTTACTGTGACATCT
GCCCAGAAGAACCAGATGGCTGTCTACCTCTGT
>BV_TRBV19*03
CACGGTGCCATCATTCAGAAACCCAAATTCCTGATCGGACTGCAAGGGCAGAAACTGACG
ATGGAATGCCAACAGACTTTTAATCACGATACCATGTACTGGTACCGGCAGGATCCAGGG
AAAGGGTTGAGACAGATCTACTATTCAATAACTGAAAAAGATATCCAGAAAGGGGAACTA
TCTGAAGGCTATAGCGTCTCTCGTGAGAAAAAGCCGTTTTTCCCTCTTACTGTGACATCT
GCCCAGAAGAACCAGATGGCTGTCTACTTCTGT
>BV_TRBV19*04
CACGGTGCCATCACTCAGAAACCCAAATTCCTGATCGGACTGCAAGGGCAGAAACTGACG
ATGGAATGCCAACAGACTTTTAATCACGATACCATGTACTGGTACCGGCAGGATCCAGGG
AAAGGGTTGAGACAGATCTACTATTCAATAACTGAAAAAGATATCCAGAAAGGGGAACTA
TCTGAAGGCTATAGCGTCTCTCGTGAGAAAAAGCCGTTTTTCCCTCTTACTGTGACATCT
GCCCAGAAGAACCAGATGGCTGTCTACCTCTGT
>BV_TRBV19*05
CACGGTGCCATCACTCAGAAACCCAAATTCCTGATCGGACTGCAAGGGCAGGAACTGACG
ATGGAATGCCAACAGGGTTTTAATCACGATGCCATGTACTGGTACCGGCAGGATCCAGGG
AAAGGGTTGAGACAGATCTACTATTCAATAGCTGAAAAAGATATCCAGAAAGGGGAACTA
TCTGAAGGCTATAGCGTCTCTCGAGAGAAAAAGCTGTTTTTCCCTCTTACTGTGACATCT
GCCCAGAAGAACCAGATGGCTGTCTACCTCTGT
>BV_TRBV20*01
GGCGCTCTTGTCGATCAATATCCCAGAAGGAGCATCTGTAGGACTGGAACTTCCATGAAG
ATTGAGTGTCACTCTGTGGGTGTTCAGGCGACTAGTGTGGCTTGGTATCGCCAGTCCCCT
CAAAGGGCCTTTGAACTGATGGCGATTTCTACTGTGAGCTCTACAGCCAAGTACGAACAA

AATTTTCCTGAGGACAAGTTTCCCATCAGTCATCCGAACTTAACCTTTTCATCTCTGACG
GTTTCAAATGCATATCCTGAAGACGGGGGCCTCTACATCTGT
>BV_TRBV20*02
GGTGCTCTTGTCGATCAATATCCCAGAAGGAGCATCTGTAGGAGTGGAACTTCCATGAAG
ATTGAGTGTCACTCTGTGGGTGTTCAGGCGACTACTGTGGCTTGGTATCGCCAGTCCCCT
CAAAGGGCCTTTGAACTGATGGCGATTTCTACTGTGAGCTCTACAGCCAAGTACGAACAA
AATTTTCCTGAGGACAAGTTTCCCATCAGTCATCCGAACTTAACCTTTTCATCTCTGACG
GTTTCAAATGCATATCCTGAAGACGGGGGCCTCTACATCTGT
>BV_TRBV20*03
GGCGCTCTTGTCGATCAATATCCCAGAAGGAGCATCTGTAGGACTGGAGCTTCCATGAAG
ATTGAGTGTCACTCTGTGGGTGTTCAGGCGACTAGTGTGGCTTGGTATCGCCAGTCCCCT
CAAAGGGCCTTTGAACTGATGGCGATTTCTACTGTGAGCTCTACAGCCAAGTACGAACAA
AATTTTCCTGAGGACAAGTTTCCCATTAGTCATCCGAACTTAACCTTTTCATCTCTGACG
GTTTCAAATGCATATCCTGAAGACGGGGGCCTCTACATCTGT
>BV_TRBV23*01
GATGCAGCAGTTACTCAGATTCCAAGACATCTGGTCAAAATGAAAGGACAGAAAGCAACG
ATGAAGTGTAGCCCTGAAAAGGGACACACTGCTGTTTACTGGTACCAGCAGAAGCAAGAC
AAAGAGTTAAAGTTCCTGATTTACTTTCAGAATAAACAGCCTCTTGATCAAATAGACATG
GTCAAGGAGCGATTCTCGGTTGAGTTTCACTCAGATACAGAGTGCAGTCTGGAAATCAAA
AGTTCGGAGGCAGGAGACTCTGCCCTGTACCTCTGT
>BV_TRBV23*02
GATGCAGCAGTTACTCAGATTCCAAGACATCTGGTCAAAATGAAAGGACAGAAAGCAACG
ATGAAGTGTAGCCCTGAAAAGGGACACACTGCTGTTTACTGGTACCAGCAGAAGCAAGAC
AAAGAGTTAAAGTTCCTGATTTACTTTCAGAATAAACAGCCTCTTGATCAAATAGACATG
GTCAAGGAGCGATTCTCGGTTGAGTTTCACTCAGATACAGAGTGCAGTCTGGAAATCAAA
AGTTCGGAGGCAGGAGACTCTGCCCTGTACCTCTGC
>BV_TRBV24*01
GATGCCAAAGTGACACAGACTCCTAGACATCTGGTCAAAGAGAAAGGACAGAAAGCAAAT
ATGATTTGTTTCCCCGAAAAAGGGCACACTGCCTTTTACTGGTATCAGCAGAGCCAAAAA
GAACTTAAACTTTTGATTAACTTCCGGAATGAAGAAATTATTGAACAAACAGACCTGGTC
AAGAAGCGATTCTCGGCTAAGTGTCCCTCAGACTCACCCTGCAGCCTGGAAATCCAGTCC
TCTGAAGCGGGAGACACAGGGTTGTACTTCTGT
>BV_TRBV24*02
GATGCCAAAGTGACACAGACTCCTAGACATCTGGTCAAAGAGAAAGGACAGAAAGCAAAT
ATGATTTGTTTCCCCGAAAAAGGGCACACTGCCTTTTACTGGTATCAGCAGAGCCAAAAA
GAACTTAAACTTTTGATTAACTTCCGGAATGAAGAAATTATTGAACAAACAGACCTGGTC
AAGAAGCGATTCTCAGCTAAGTGTCCCTCAGACTCACCCTGCAGCCTGGAAATCCAGTCC
TCTGAAGCGGGAGACACAGGGTTGTACTTCTGT
>BV_TRBV26*01
AATGCCAAAGTCATCCAGACTCCAAGATATCTGACCAAAGGGAAAGAACAGAAAGCAAAG
ATGAGCTGTCGCCCTGAAAAGGGCCACGCTGTTGTGCTCTGGTATAAACAGAATAACAAC
AATGAATTTACATTCTTGATTAGCTTTCAGAATCAAGACGTTCTTCAACAAATAGACATG
GTCAAGAATCGATTCTCAGCTGAGTGTCCTTCGAACTCACCTTGCAGCCTGGAAATCCAG
TCCTCTGAGGCGGGAGACTCAGCACTGTACTTCTGT
>BV_TRBV26*02
AATGCCAAAGTCATCCAGACTCCAAGATATCTGACCAAAGGGAAAGAACAGAAAGCAAAG
ATGAGCTGTCGCCCTGAAAAGGGCCACGCTGTTGTGCTCTGGTATAAACAGAATAACAAC
AATGAATTTACATTCTTGATTAGCTTTCAGAATCAAGACGTTCTTCAACAAATAGACATG
GTCAAGAATCGATTCTCAGCTGAGTGTCCTTCGAACTCACCTTGCAGCCTGGAAATCCAG
TCCTCCGAGGCGGGAGACTCAGCACTGTACTTCTGT
>BV_TRBV29*01
GACATGGACGTAACCCAGACACCAAGATATCTGGTCAAAAGAACGGGAGAGGAAGTTTTG

CTGGACTGTGAACAGTATATGAGCCATGAAAGAATGTTCTGGTATCGACAAGACCCAGGT
CTAGGGCTACGGTTGATTCATTTTTCATATGATGTTGACAGTAACAGCGAAGGAGACATC
CCTGAAGGATACAGGGTCTCTCGGAAGAAGCGGGAGCGTTTCTCCCTGATTCTGGAGTCT
GCAAAAACCAGTCAGACATCTGTGTACTTCTGT
>BV_TRBV29*02
GACATGGACATAACCCAGACACCAAGATATCTGGTCAAAAGAACGGGAGAGGAAGTTTTG
CTGGACTGTGAACAGTATATGAGCCATGAAAGAATGTTCTGGTATCGACAAGACCCAGGT
CTAGGGCTACGGTTGATTCATTTTTCATATGATGTTAACAGTAACAGCGAAGGAGACATC
CCTGAAGGATACAGGGTCTCTCGGAAGAAGCGGGAGCGTTTCTCCCTGATTCTGGAGTCT
GCAAAAACCAGCCAGACATCTGTGTACTTCTGT
>BV_TRBV30*01
AGTGTCCTCGTCTCCCAAAAGCCCAAAAGGGACATCTGTCAAAGTGGAACCTCGCTGAAA
ATCCAGTGTGTGCTTGACAGTCAAGTTGCTGGGATGTTCTGGTACAGACAGATCCCGGGA
CAGAGCTTGGTGCTCATGGCAGCTGCAAATCAAGGCTCAGAAGCCACATATGAGAAAGAA
TTCGACAAGGATAAGTTTCCTATCAGCCGCCCAAACCTAACATTCTCCACGTTGACAGTG
AGCAATGCGAGGCCTGAAGACAGCAGCTTCTATTTCTGC
>BV_TRBV30*02
AGTGTCCTCGTCTCCCAAAAGCCCAAAAGGGCCATCTGTCAAAGTGGAACCTCGCTGAAA
ATCCAGTGTGTGCTTGACAGTCAAGTTGCTGGGATGTTCTGGTACAGACAGATCCCGGGA
CAGAGCTTGGTGCTCATGGCAGCTGCAAATCAAGGCTCAGAAGCCACATATGAGAAAGAA
TTCGACAAGGATAAGTTTCCTATCAGCCGCCCAAACCTAACATTCTCCACATTGACAGTG
AGCAATGCGAGGCCTGAAGACAGCAGCTTCTATTTCTGC
>BV_TRBV30*03
AGTGTCCTCGTCTCCCAAAAGCCCAAAAGGGACATCTGTCAAAGTGGAACCTCGCTGAAA
ATCCAGTGTGTGCTTGACAGTCAAGTTACAGGGATGTTCTGGTACAGACAGATCCCGGGA
CAGAGCTTGGTGCTCATGGCAGCTGCAAATCAAGGCTCAGAAGCCACGTATGAGAAAGAA
TTCGACAAGGACAAGTTTCCTATCAGCCGCCCAAACCTAACATTCTCCACGTTGACAGTG
AGCAATGCGAGGCCTGAAGACAGCAGCTTCTATTTCTGC
>BV_TRBV30*04
AGTGTCCTCGTCTCCCAAAAGCCCAAAAGGGACATCTGTCAAAGTGGAACCTCGCTGAAA
ATCCAGTGTGTGCTTGACAGTCAAGTTGCTGGGATGTTCTGGTACAGGCAGATCCCGGGA
CAGAGCTTGGTGCTCATGGCAGCTGCAAATCAAGGCTCAGAAGCCACGTATGAGAAAGAA
TCCGACAAGGATAAGTTTCCTATCAGCCGCCCAAACCTAACATTCTCCACGTTGACAGTG
AGCAATGCGAGGCCTGAAGACAGCAGCTTCTATTTCTGC
>BV_TRBV31*01
GCTCAGATTATCCACCAACAGCCAGCTGTCAGTGTGCAGCCTGTGGGCAGTTCACTCTCC
GTGGAGTGTACCGTCCAGGGACAATCAAGCCCCAACCTCTACTGGTACTGGCAGGACAAA
GGAGGGACCCTGCAGCAGCTCTTTTACTCTATTAGTGTTGGTGATGTGGTCTCGGTGGCA
GCTCGGAACCTCTCGGCCTCCAGGCCCCAGGATGGCCAGTTCACCCTGAGCTCTGAGAAG
CTGCTTCTCAGTCACTCTGGCTTCTACTTCTGT
>BV_TRBV31*02
GCTCAGATTATCCACCAACAGCCAGCTGTCAGTGTGCAGCCTGTGGGCAGTTCACTCTCT
GTGGAGTGTACCGTCCAGGGACAATCAAGCCCCAACCTCTACTGGTACTGGCAGGACAAA
GGAGGGACCCTGCAGCAGCTCTTTTACTCTATTAGTGTTGGTGATGTGGTCTCAGTGGCA
GCTCGGAACCTCTCAGCCTCCAGGCCCCGGGATGGCCAGTTCATCCTGAGCTCTGAGAAG
CTGTTTCTCAGTCACTCTGGCTTCTACTTCTGT
>BV_TRBV31*03
GCTCAGATTATCCACCAACAGCCAGCTGTCAGTGTGCAGCCTGTGGGCAGTTCACTCTCT
GTGGAGTGTACCGTCCAGGGACAATCAAGCCCCAACCTCTACTGGTACTGGCAGGACAAA
GGAGGGACCCTGCAGCAGCTCTTTTACTCTATTAGTGTTGGTGATGTGGTCTCAGTGGCA
GCTCGGAACCTCTCAGCCTCCAGGCCCCGGGATGGCCAGTTCACCCTGAGCTCTGAGAAG
CTGCTTCTCAGTCACTCTGGCTTCTACTTCTGT

>BV_TRBV31*04
GCTCAGATTATCCACCAACAGCCAGCTGTCAGTGTGCAGCCTGTGGGCAGTTCACTCTCT
GTGGAGTGTACCGTCCAGGGACAATCAAGCCCCAACCTCTACTGGTACTGGCAGGACAAA
GGAGGGACCCTGCAGCAGCTCTTTTACTCTATTAGTGTTGGTGATGTGGTCTCAGTGGCA
GCTCGGAACCTCTCAGCCTCCAGGCCCCGGGATGGCCAGTTCATCCTGAGCTCTGAGAAG
CTGCTTCTCAGTCACTCTGGCTTCTACTTCTGT
>BV_TRBV40*01
GAGGCTCAAGTCACCCAGGCTCCAAGACATCTTATCACAGAGACTGGGAAGAAGTTGATA
ATAACTTGCTCCCAGAATATGAATCATGATTATATGTTCTGGTATAGACAAGACTCAGGC
CTGGGTCTAAAGCTGATCTATTACTCAATTAATGCTGATCATGTCGATAAAGGAGAAGTC
TCTGATGGGTATAGTGTCTCTAGAGAAGACAAAAAAGCCTTTCTTCTGACACTGGAATCT
ACCAGAGTCAGCCAGACCTCCTTGTACCTCTGT
>BV_TRBV40*02
GAGGCTCAAGTCACCCAGGCTCCAAGACATCTTATCACAGAGACTGGGAAGAAGTTGATA
ATAACTTGCTCCCAGAATATGAATCATGATTATATGTTCTGGTATAGACAAGACTCAGGC
CTGGGTCTAAAGCTGATCTATTACTCAATTAATGCTGATCATGTCGATAAAGGAGAAGTC
TCTGATGGGTATAGTGTCTCTAGAGAAGACAAAAAAGCCTTTCTTCTGACACTGGAATCT
ACCAGAGTCAGCCAAACCTCCTTGTACCTCTGC
>BV_TRBV40*03
GAGGCTCAAGTCACCCAGGCTCCAAGACACCTCATCACAGAGACTGGGAAGAAGTTGATA
ATAACTTGCTCCCAGAATATGAATCATGATTATATGTTCTGGTATAGACAAGACTCAGGC
CTGGGTCTAAAGCTGATCTATTACTCAATTAATGCTGATCATGTCGATAAAGGAGAAGTC
TCTGATGGGTATAGTGTCTCTAGAGAAGACAAAAAAGCCTTTCTTCTGACACTGGAATCT
ACCAGAGTCAGCCAAACCTCCTTGTACCTCTGC
>BV_TRBV40*04
GAGGCTCAAGTCACCCAGGCTCCAAGACACCTCATCACAGAGACTGGGAAGAAGTTGATA
ATAACTTGCTCCCAGAATATGAATCATGATTATATGTTCTGGTATAGACAAGACTCAGGC
CTGGGTCTAAAGCTGATCTATTACTCAATTAATGCTGATCATGTCGATAAAGGAGAAGTC
TCTGATGGGTATAGTGTCTCTAGAGAAGACAAAAAAGCCTTTCTTCTGACACTGGAATCT
ACCAGAGTCAGCCAAACTTCCTTGTACCTCTGC
>BV_TRBV40*05
GAGGCTCAAGTCACCCAGGCTCCAAGACATCTTATCACAGAGACTGGGAAGAAGTTGATA
ATAACTTGCTCCCAGAATATGAATCATGATTATATGTTCTGGTATAGACAAGACTCAGGC
CTGGGTCTAAAGCTGATCTATTACTCAATTAATGCTGATCATGTCGATAAAGGAGAAGTC
TCTGATGGGTATAGTGTCTCTAGAGAAGACAAAAAAGCCTTTCTTCTGACACTGGAATCT
ACCAGAGTCAGCCAAACTTCCTTGTACCTCTGT
>BV_TRBV41*01
AAGGCTGGAGTCTCCCAGTTTCCCGCTCACACTATCATCAAAAAGGGACAGAATGTATCT
TTCAGGTGCGATCCAATTTCAGGTCACACGCGCCTTTTCTGGTATCAGCAGAAATCGGGG
CAAGGCCCAGAGTTTCTGACTTATTTCCAGAATGAAGTCGCACCAGACAAATCAGGGCTG
CCTGGAGATCGCTTCTCTGCAGAGAGGCCTGATGGATCCTACTCTGTTCTGAAGATCCAG
CCTGCGAAGCGGGAGGACTCGGCGGTGTACCTCTGT
>BV_TRBV41*02
AAGGCTGGAGTCTCCCAGTTTTCCGCTCACACTATCATCAAAAAGGGACAGAATGTATCT
TTCAGGTGCGATCCAATTTCAGATCACACGCGCCTTTTCTGGTATCAGCAGAAATCGGGG
CAAGGCCCAGAGTTTCTGACTTATTTCCAGAATGAAGTCGCACCAGACAAATCAGGGCTG
CCTGGAGATCGCTTCTCTGCAGAGAGGCCTGATGGATCCTACTCTGTTCTGAAGATCCAG
CCTGCGAAGCAGGAGGACTCGGCGGTGTACCTCTGC
>BV_TRBV41*03
AAGGCTGGAGTCTCCCAGTTTCCCGCTCACACTATCATGAAAAAGGGACAGAATGTATCT
TTCAGGTGCGATCCAATTTCAGGTCACCCGCACCTTTTCTGGTATCAGCAGAAATCGGGG
CAAGGCCCAGAGTTTCTGACTTATTTCCAGAATGAAGTCGCACCAGACAAATCAGGGCTG

CCTGGAGATCGCTTCTCTGCAGAGAGGCCTGATGGATCCTACTCTGTTCTGAAGATCCAG
CCTGCGAAGCGGGAGGACTCGGCCGTGTACCTCTGC
>BV_TRBV41*04
AAGGCTGGAGTCTCCCAGTTTCCCGCTCACACTATCATCAAAAAGGGACAGAATGTATCT
TTCAGGTGCTATCCAATTTCAGGTCACACCCGCCTTTTCTGGTATCAGCAGAAATCGGGG
CAAGGCCCAGAGTTTCTGACTTATTTCCAGAATGAAGTCACACCAGACAAATCAGGGCTG
CCTGGAGATCGCTTCTCTGCAGAGAGGCCTGATGGATCCTACTCTGTTCTGAAGATCCAG
CCTGCGAAGCGGGAGGACTCGGCGGTGTACCTCTGC
>BV_TRBV41*05
AAGGCTGGAGTCTCCCAGTTTCCCGCTCACACTATCATCAAAAAGGGACAGAATGTATCT
TTCAGGTGCTATCCAATTTCAGGTCACACCCGCCTTTTCTGGTATCAGCAGAAATCGGGG
CAAGGCCCAGAGTTTCTGACTTATTTCCAGAATGAAGTCGCACCAGACAAATCAGGGCTG
CCTGGAGATCGCTTCTCTGCAGAGAGGCCTGATGGATCCTACTCTGTTCTGAAGATCCAG
CCTGCGAAGCGGGAGGACTCGGCGGTGTACCTCTGC
>BV_TRBV42*01
GATGGACAAGTTATCCAGATTCCAAAACATCTATTCAAAGGGAAAGGACAAAAAGCAAAG
ATGAGCTGTATCCCTGAAAACGGACATACTTATGTTTATTGGTACCAACAGAAGCAGAAT
AAAGAGTTGAAATTTTTGATTTACTTTCAACGTCAAGAAGTTCGTGAAAGAATAGACTTT
GTTAAGGAGCGATTTGTAGCTGAGTGTCCCATAAACTCGCCCTGCAGCCTGGAAATCCAG
TTCTCCGAGGCAGGAGACTCAGCACTGTACCTCTGT
